# Centromere Interactions Promote the Maintenance of the Multipartite Genome in Agrobacterium tumefaciens

**DOI:** 10.1128/mbio.00508-22

**Published:** 2022-05-10

**Authors:** Zhongqing Ren, Qin Liao, Ian S. Barton, Emma E. Wiesler, Clay Fuqua, Xindan Wang

**Affiliations:** a Department of Biology, Indiana University, Bloomington, Indiana, USA; Max Planck Institute for Terrestrial Microbiology

**Keywords:** *Agrobacterium tumefaciens*, ParB, RepB, PopZ, PodJ, GPR, Hi-C, ChIP-seq

## Abstract

Many pathogens or symbionts of animals and plants contain multiple replicons, a configuration called a multipartite genome. Multipartite genomes enable those species to replicate their genomes faster and better adapt to new niches. Despite their prevalence, the mechanisms by which multipartite genomes are stably maintained are poorly understood. Agrobacterium tumefaciens is a plant pathogen that contains four replicons: a circular chromosome (Ch1), a linear chromosome (Ch2), and two large plasmids. Recent work indicates that their replication origins are clustered at the cell poles in a manner that depends on their ParB family centromeric proteins: ParB1 for Ch1 and individual RepB paralogs for Ch2 and the plasmids. However, understanding of these interactions and how they contribute to genome maintenance is limited. By combining genome-wide chromosome conformation capture (Hi-C) assays, chromatin-immunoprecipitation sequencing (ChIP-seq), and live cell fluorescence microscopy, we provide evidence here that centromeric clustering is mediated by interactions between these centromeric proteins. We further show that the disruption of centromere clustering results in the loss of replicons. Our data establish the role of centromeric clustering in multipartite genome stability.

## INTRODUCTION

Many species across the bacterial kingdom contain a multipartite genome (1). It has been proposed that a multipartite genome imparts to these bacteria multiple competitive advantages to adapt to new niches, such as faster genome duplication, more rapid growth, and more flexible gene dosage regulation through modulating the copy number of individual replicons (1, 2). However, a multipartite genome poses challenges for genome stability during cell proliferation, especially for the replicons that are not essential for every growth condition. The mechanisms by which multipartite genomes are maintained are still unknown.

**FIG 1:**
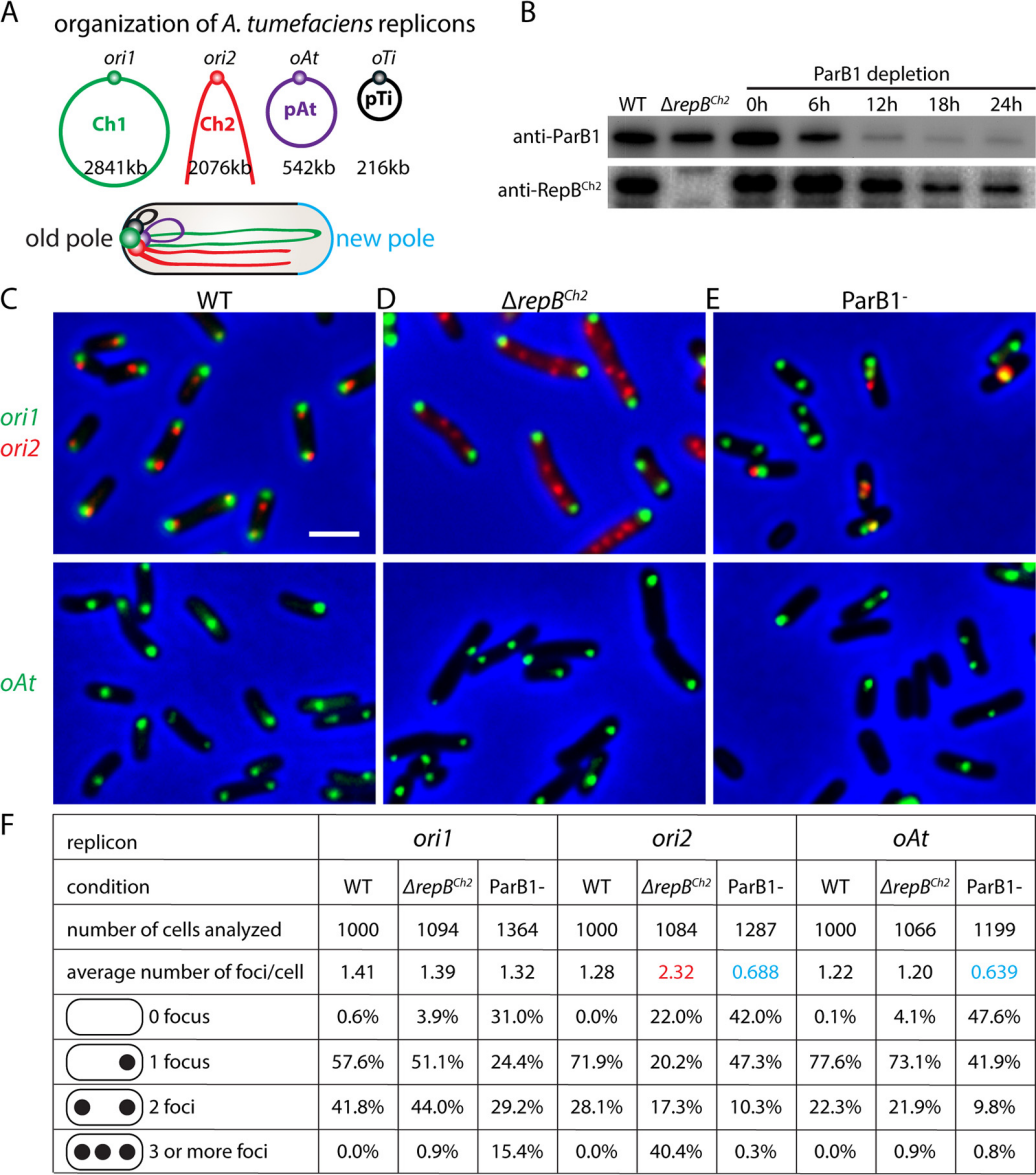
Centromeric proteins ParB1 and RepB^Ch2^ are important for genome maintenance. (A) Schematic model of the multipartite genome of A. tumefaciens and its cellular organization. In a newborn cell, the four origins are clustered together at the old cell pole, opposite the new pole (light blue). The arms of Ch1 and Ch2 are aligned along the cell length. (B) Immunoblot analysis of ParB1 depletion time course matching microscopy experiments. The levels of ParB1 and RepB^Ch2^ are shown. ParB1 depletion strain (AtWX192) (11) contains *PtraI-riboswitch-parB1* at the *tetRA* locus and *parB1* deleted from the endogenous locus. The presence of inducers 1 μM acylhomoserine lactone (AHL) and 2 mM theophylline allows expression of ParB1. Cells were first grown in ATGN solid medium or liquid medium containing inducers. To deplete ParB1, cells were washed in ATGN medium 4 times and then diluted into fresh ATGN without inducers. Cultures were diluted before their optical density at 600 nm (OD_600_) reached 0.6 to prevent cells from entering stationary phase. Samples were taken at the indicated time points. (C to E) The localization of origins in the wild type (top, AtWX356; bottom, AtWX359) (C) or *ΔrepB^Ch2^* (top, AtWX402; bottom, AtWX500) mutant (D) or after 24 h of ParB1 depletion (top, AtWX496; bottom, AtWX498) (E). The origins are labeled using *mcherry-parB^P1^-parS^P1^* at a position 50 kb away from *ori1* (green, top) or *ygfp-parB^pMT1^-parS^pMT1^* 57 kb away from *ori2* (red, top) or 11 kb away from *oAt* (green, bottom). Images of full time course of ParB1 depletion can be found in Fig. S1. Pseudocolors were assigned as indicated. Scale bar represents 2 μm. (F) Analysis of origin number in WT or *ΔrepB^Ch2^* strain or ParB1 depletion for 24 h. Images were analyzed using MetaMorph software. Red and blue colors highlight numbers that are higher or lower, respectively, than those for the WT.

Agrobacterium tumefaciens, the causative agent of crown gall disease on plants, has four replicons: a circular chromosome (Ch1, 2,841 kb), a linear chromosome (Ch2, 2,076 kb), and two plasmids, pAt (542 kb) and pTi (216 kb) (3, 4) (Fig. 1A). Like many other species, the primary chromosome (Ch1) replicates first, followed by the replication of secondary replicons (Ch2 and plasmids) (5–10). Our recent study (11) characterized the organization of this multipartite genome and revealed that the origins of the four replicons are clustered together and localized at the two cell poles (Fig. 1A). Moreover, the arms of Ch1 and Ch2 are aligned not only within each chromosome to generate interarm interactions but also between the two chromosomes, resulting in Ch1-Ch2 alignment (Fig. 1A). It was found that the centromeric proteins, ParB1 for Ch1 and RepB^Ch2^ for Ch2, are required for origin clustering and Ch1-Ch2 alignment (11). Despite these known interactions, it is unclear how origin clustering contributes to multipartite genome maintenance and whether origin clustering is through direct interactions among the centromeric proteins or mediated by other factors.

A. tumefaciens has a unipolar growth mechanism (12) in which the cell wall is only synthesized at the new cell pole, also called the growth pole. There are three proteins (GPR, PopZ, and PodJ) that are thought to serve as polar organizing proteins. At the growth pole, GPR and PopZ are both required for unipolar growth, cell shape, and normal cell division (13–18). Moreover, in the absence of PopZ, the origin of Ch1 (or *ori1*) disassociates from the new pole, indicating that PopZ anchors the origin to the new pole (14), likely through interaction with ParB1 protein (19). The cell pole opposite the growth pole is referred to as the old pole. A third polar landmark protein, PodJ, localizes to the old pole and plays an essential role in the growth pole-to-old pole transition (13, 20). It is unclear whether these polar organizers play a role in clustering the origins together.

In several bacterial species, it has been shown that polar factors interact with sequence-specific DNA-binding proteins to anchor a chromosomal region to the cell pole. For instance, in Caulobacter crescentus, PopZ polymerizes into a matrix at the cell pole, directly interacts with the centromeric ParB/*parS* complex, and anchors the chromosome origin region at the pole (19, 21, 22). Similarly, in sporulating Bacillus subtilis, DivIVA nucleates at the cell pole and localizes RacA-bound origin-proximal RAM sites to the cell poles (23–25). In another example, the Vibrio cholerae polar protein HubP has also been suggested to interact with the centromeric ParB protein and localize its origin to the pole (26). In A. tumefaciens, since the replication origins of the four replicons are all localized to the cell poles (5, 10, 11) (Fig. 1A), it is possible that certain polar factors interact with each replication origin individually and serve as a platform to cluster the origins together.

In this study, we found that disruption of origin clustering led to the loss of secondary replicons in A. tumefaciens, establishing that origin clustering is critical for the maintenance of the multipartite genomes in bacteria. We further investigated the molecular mechanism of the origin clustering. We found that although the three polar organizers PopZ, PodJ, and GPR were important for the polar localization of the origins, they were not required for the clustering of origins. Furthermore, we showed that the centromeric proteins ParB1 and RepB^Ch2^ interacted when expressed in a distantly related bacterium, B. subtilis. Altogether, we posit that origin clustering is mediated through direct interactions between different centromeric proteins and is critical for maintaining the integrity of this multipartite genome.

## RESULTS

### Origin clustering is important for maintaining the secondary replicons.

To test how the centromeric proteins contribute to the replication and segregation of the replicons, we visualized the replication origins in the absence of ParB1 or RepB^Ch2^. To label the origins in live cells, we inserted a visualization cassette, *mcherry-parB^P1^-parS^P1^*, at a position 50 kb away from *ori1*, or *ygfp-parB^pMT1^-parS^pMT1^*, 57 kb away from *ori2* or 11 kb away from *oAt* (11, 27). Each cassette contains yGFP or mCherry fused to ParB from the pMT1 or P1 plasmid and the *parS* site of pMT1 or P1, respectively (28). When inserted into the A. tumefaciens genome, these cassettes enable the visualization (Fig. 1C to E) and enumeration (Fig. 1F) of the loci of interest in live cells but do not interfere with the function of endogenous ParB/RepB proteins or the organization or the segregation of the gene loci (11). We note that we were not able to track the pTi plasmid in this analysis because pTi was lost when we constructed the ParB1 depletion strain in two independent attempts (11). In Fig. 1C to E, *ori1* and *ori2* were visualized in the same cells at the top, and *oAt* was visualized on the bottom. In the wild-type cells, the origins of the chromosomes and plasmids are present as one or two copies localized to either the old cell pole or both cell poles (11) (Fig. 1C). In the Δ*repB^ch2^* markerless deletion mutant (Fig. 1B) there were more copies of *ori2*, which were randomly distributed in the cell, in contrast to the bipolar localization (Fig. 1D and F). Similarly, when the essential ParB1 protein was depleted by removing inducers (Fig. 1B), *ori1* showed aberrant copy number and loss of polar localization (Fig. 1E and F; see also Fig. S1 in the supplemental material). Thus, ParB1 and RepB^Ch2^ are important for their individual replicon’s segregation and replication. These data are consistent with the function of ParB family proteins found in other species (29–32).

10.1128/mbio.00508-22.1 FIG S1 ParB1 is important for genome maintenance. Visualization of origin localization when ParB1 was depleted for the indicated durations in AtWX496 (top) and AtWX498 (bottom). The origins are labeled using *mcherry-parB^P1^-parS^P1^* 50 kb away from *ori1* (green), top, or *ygfp-parB^pMT1^-parS^pMT1^* at 57 kb away from *ori2* (red, top) or 11 kb away from *oAt* (green, bottom). Pseudocolors were assigned as indicated. Protein levels can be found in Fig. 1B. Scale bar represents 2 μm. ParB1 depletion was performed by washing away the inducers (1 μM AHL and 2 mM theophylline). Download FIG S1, PDF file, 2.1 MB. Copyright © 2022 Ren et al. 2022 Ren et al. https://creativecommons.org/licenses/by/4.0/ This content is distributed under the terms of the Creative Commons Attribution 4.0 International license.

Next, we analyzed whether the absence of RepB^Ch2^ or ParB1 affected the replication and segregation of other replicons. In the Δ*repB^ch2^* mutant, the copy number and the localization of *ori1* and *oAt* were very similar to those of the WT (Fig. 1D and F), suggesting that RepB^Ch2^ does not affect the replication or segregation of other replicons. However, and strikingly, in the absence of ParB1, the average numbers of *ori2* and *oAt* per cell were reduced dramatically (Fig. 1E and F), with >40% of cells having no foci for these origins, indicating the loss of Ch2 and pAt. We note that the pTi plasmid was already lost in our ParB1 depletion strain, likely due to diminished ParB1 levels at certain steps of strain construction (11). These experiments showed that RepB^Ch2^ is only important for the maintenance of Ch2, but ParB1 is important for the maintenance of all four replicons. Since ParB1 does not bind to Ch2 or the plasmids in our chromatin-immunoprecipitation sequencing (ChIP-seq) experiments but is required to cluster all the replicons together (11), we reasoned that the effect of ParB1 depletion on these secondary replicons was due to the loss of clustering between the origins (11). Thus, while each centromeric protein is important for the segregation of its cognate replicon, clustering with the origin of Ch1 is critical for the maintenance of secondary replicons.

### Polar organizers are important for origin polar localization.

Next, we set out to test the mechanism of centromeric clustering. Since it has been shown in several other species that polar factors interact with sequence-specific DNA-binding proteins to anchor a chromosomal region to the cell pole, such as PopZ in C. crescentus (19, 21, 22), DivIVA in B. subtilis (23–25), and HubP in V. cholerae (26), we investigated whether the three known pole organizing proteins in A. tumefaciens, PodJ, PopZ, and GPR, serve as a bridge for the interreplicon contacts.

**FIG 2:**
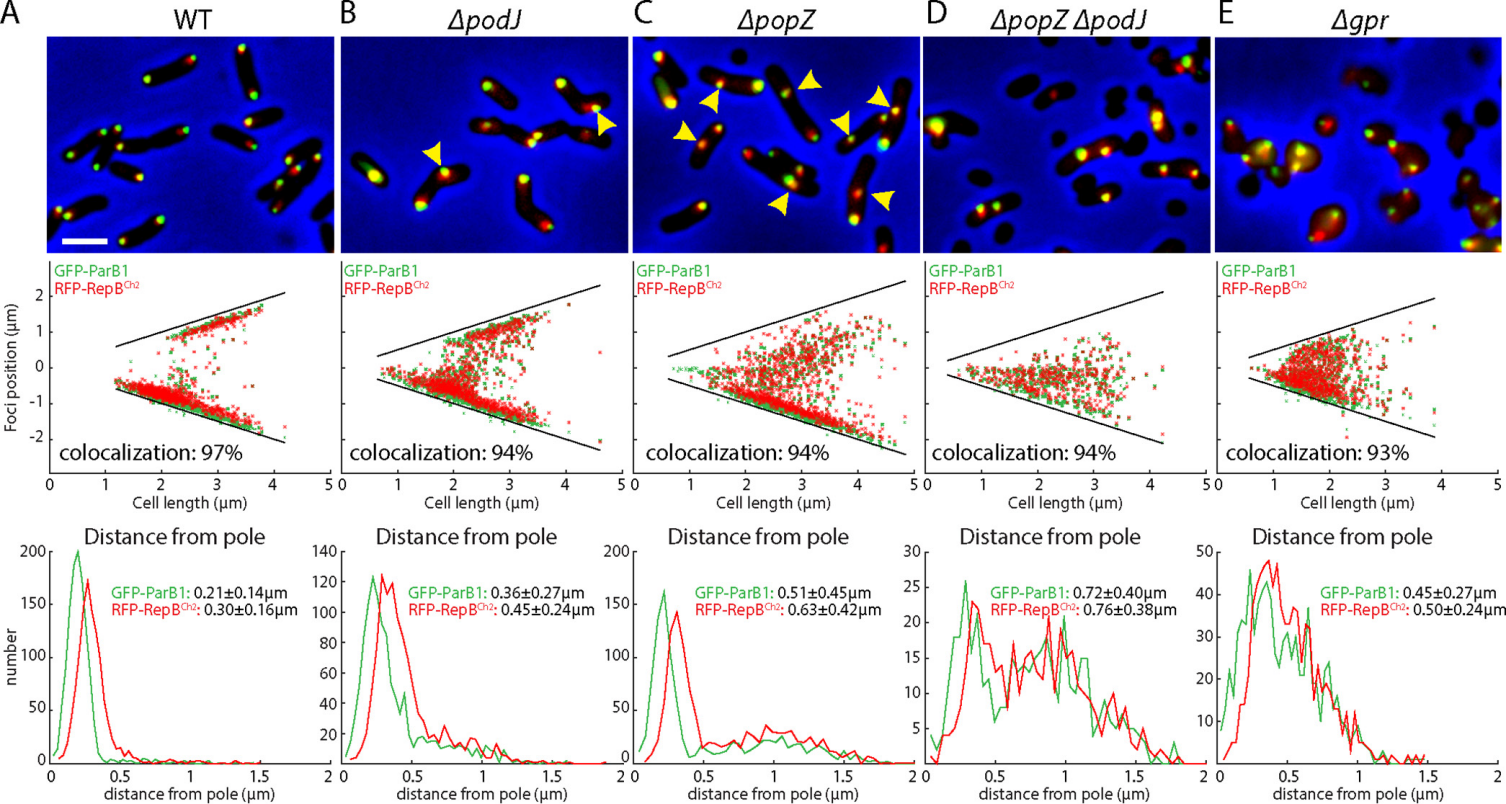
Polar organizers are required for polar localization of the origins. (A to E) Visualization of *ori1* (green) and *ori2* (red) in WT (AtWX263) (A) and indicated *ΔpodJ* (AtWX307) (B) *ΔpopZ* (AtWX303) (C), *ΔpopZ ΔpodJ* (AtWX305) (D), and *Δgpr* (AtW309) (E) mutants. *ori1* and *ori2* are labeled using GFP-ParB1 and RFP-RepB^Ch2^ expressed from a single pSRKKm-based plasmid. (Top) Cropped fluorescence microscopy images. (Middle) Plots of focus positions. (Bottom) Plots of distance of foci from the nearest pole. Scale bar represents 2 μm. Yellow carets point to the foci that are distant from the cell pole. Colocalization was defined as a pair of green and red foci that are within an interfocal distance of fewer than 6 pixels. Detailed image analysis can be found in Fig. S2 and Materials and Methods.

We succeeded in making markerless deletions of *ΔpodJ*, *ΔpopZ*, or Δ*gpr* in strain C58 growing in minimal ATGN medium (33) (Fig. S2A). Consistent with earlier studies (14–17, 20), in the absence of these polar organizer proteins, cells showed various defects in cell shape and cell division, such as ectopic cell poles (Fig. 2), broader distribution of cell length (Fig. S2G), and increased numbers of anucleate cells (Fig. S2I); Δ*gpr* mutant lost its rod shape and became spherical (Fig. 2E and Fig. S2H). We note that the defects we observed here in minimal ATGN medium were milder than those previously reported in LB (15, 17, 20). We suspect the discrepancy was due to the medium difference and slower growth (Fig. S2A) and not suppressor mutations, because we performed whole-genome sequencing (WGS) on all these mutants that did not reveal any additional mutations.

10.1128/mbio.00508-22.2 FIG S2 Polar organizers are required for polar localization of the origins. (A) Tenfold serial dilutions of the indicated strains spotted on ATGN plate (left) or LB plate (right). (B to F) Plots of the localization of *ori1* (green) and *ori2* (red) in WT (AtWX263) (B), *ΔpodJ* (AtWX307) (C), *ΔpopZ* (AtWX303) (D), *ΔpopZ ΔpodJ* (AtWX305) (E), *Δgpr* (AtW309) (F) strains for cells containing a single *ori1* and *ori2* focus (top) or two *ori1* and *ori2* foci (bottom). The percentage of cells in these subpopulations can be found in panel H. (G and H) Distribution of cell length (G) and cell width (H) in the indicated strains. (I) Image analysis. Colocalization was defined as a pair of green and red foci that are have an interfocal distance of fewer than 6 pixels. Images were analyzed using Oufti software (see Materials and Methods). Download FIG S2, PDF file, 2.1 MB. Copyright © 2022 Ren et al. 2022 Ren et al. https://creativecommons.org/licenses/by/4.0/ This content is distributed under the terms of the Creative Commons Attribution 4.0 International license.

To test whether PodJ, PopZ, and GPR are required for the proper localization of the replication origins, we visualized *ori1* and *ori2* in live cells by expressing GFP-ParB1 and RFP-RepB^Ch2^, respectively. We quantified the distance between an origin focus and the cell pole. In the WT, regardless of whether the cells had one or two copies of origins, both *ori1* and *ori2* were localized at the tips of the cell poles (Fig. 2A and Fig. S2B). In *ΔpodJ* cells with a single copy of *ori1* and *ori2*, the origins were away from the old pole (Fig. 2B and Fig. S2C). Interestingly, in *ΔpodJ* cells with two copies of origins, one copy was away from the pole but the other origin was close to the pole (Fig. 2B, yellow caret, and Fig. S2C). In *ΔpopZ* cells with one copy of *ori1* and *ori2*, the origins were still localized to the old pole in most cells (Fig. 2C and Fig. S2D). However, in *ΔpopZ* cells with two copies of origins, one copy was close to the pole but the other copy was distant from the pole (Fig. 2C, yellow caret, and Fig. S2D). These data are consistent with the idea that PodJ is required to localize the origins to the old pole but not to the new pole (10), and PopZ is required to anchor the origin but only to the new pole (10, 14). In the *ΔpopZ ΔpodJ* double mutant, the origins were no longer localized to either pole (Fig. 2D and Fig. S2E). Finally, in the *Δgpr* mutant, the cells became spherical and localization of the origins appeared random (Fig. 2E and Fig. S2F). These results indicate that polar organizers PodJ, PopZ, and GPR are important for the localization of the origins.

To test whether PopZ, PodJ, and GPR effects on origin polar localization are through interacting with the DNA in a complex, we analyzed the genome-wide enrichment of GFP-PopZ, GFP-PodJ, and GFP-GPR using anti-GFP ChIP-seq (Fig. S3). We observed that the enrichment of GFP-PodJ or GFP-GPR at *ori1* and *ori2* regions was barely above the background levels, but GFP-PopZ was strongly enriched at these regions (Fig. S3A to C). Previous findings in C. crescentus showed that PopZ directly interacts with ParB and anchors the origins to the cell pole (19). To test whether GFP-PopZ enrichment is dependent on ParB1 or RepB^Ch2^, we performed ChIP-seq in the absence of these proteins. When ParB1 was depleted, GFP-PopZ enrichment at *ori1* was abolished and that at *ori2* was reduced to 35.9% (Fig. S3D). In the *ΔrepB^Ch2^* mutant, GFP-PopZ enrichment at *ori1* was unchanged but at *ori2* was absent (Fig. S3D). These results suggest that PopZ interacts with ParB1 and to a much lesser degree with RepB^Ch2^, and it anchors the origins to the pole, while GPR and PodJ affect polar localization of the origins indirectly, perhaps by maintaining cell shape or by positioning other unknown origin-anchoring proteins at the cell pole.

10.1128/mbio.00508-22.3 FIG S3 PopZ is enriched at *ori1* and *ori2*, which require ParB1 and RepB^Ch2^, respectively. (A to C) ChIP enrichment (ChIP/input) of GFP-PopZ (AtWX234) (A), GFP-PodJ (AtWX263) (B), and GFP-GPR (AtWX236) (C). Whole-genome profiles are shown on the left in 1-kb bins, and high-resolution plots of *ori1* and *ori2* regions are shown on the right in 100-bp bins. Black asterisks indicate an enrichment peak present in all of our anti-GFP ChIP-seq experiments regardless of the fusion protein. Blue and gray dotted lines indicate the *parS1* and *parS2* sites, respectively. (D) High-resolution ChIP enrichment (ChIP/input) of GFP-PopZ at *ori1* (top) and *ori2* (bottom) in ParB1^+^ (AtWX289 with inducers 1 μM AHL and 2 mM theophylline), ParB1 (AtWX289 without inducers), and Δ*repB^Ch2^* (AtWX291) strains. The enrichment of GFP-PopZ at the *ori1* and *ori2* regions depends on the presence of ParB1 and RepB^Ch2^, respectively. Download FIG S3, PDF file, 0.9 MB. Copyright © 2022 Ren et al. 2022 Ren et al. https://creativecommons.org/licenses/by/4.0/ This content is distributed under the terms of the Creative Commons Attribution 4.0 International license.

### Polar organizers are not required for the clustering of origins.

While PopZ, PodJ, and GPR are important for the localization of replicon origins, we next analyzed whether these proteins are required for origin clustering. We first visualized and quantified the colocalization of *ori1* and *ori2*. Qualitatively, in our micrographs of WT and mutants, the *ori1* and *ori2* foci were largely colocalized regardless of the mutations tested (Fig. 2). Quantitatively, we measured the distance between the centers of green (*ori1*) and red (*ori2*) foci. In our images, the average diameter of a fluorescence focus was 6 pixels. When the red and green foci were overlapping, i.e., had an interfocal distance of less than 6 pixels, we defined these as colocalizing. Using this criterion, we found that in the WT, 97% of *ori1* foci were colocalized with an *ori2* focus (Fig. S2I). In Δ*podJ*, *ΔpopZ*, *ΔpopZ ΔpodJ*, and *Δgpr* strains, 94%, 94%, 94%, and 93% of *ori1* foci were colocalized to *ori2* (Fig. S2I). These results indicate that polar organizers are not required for the colocalization of *ori1* and *ori2* in the cell.

**FIG 3:**
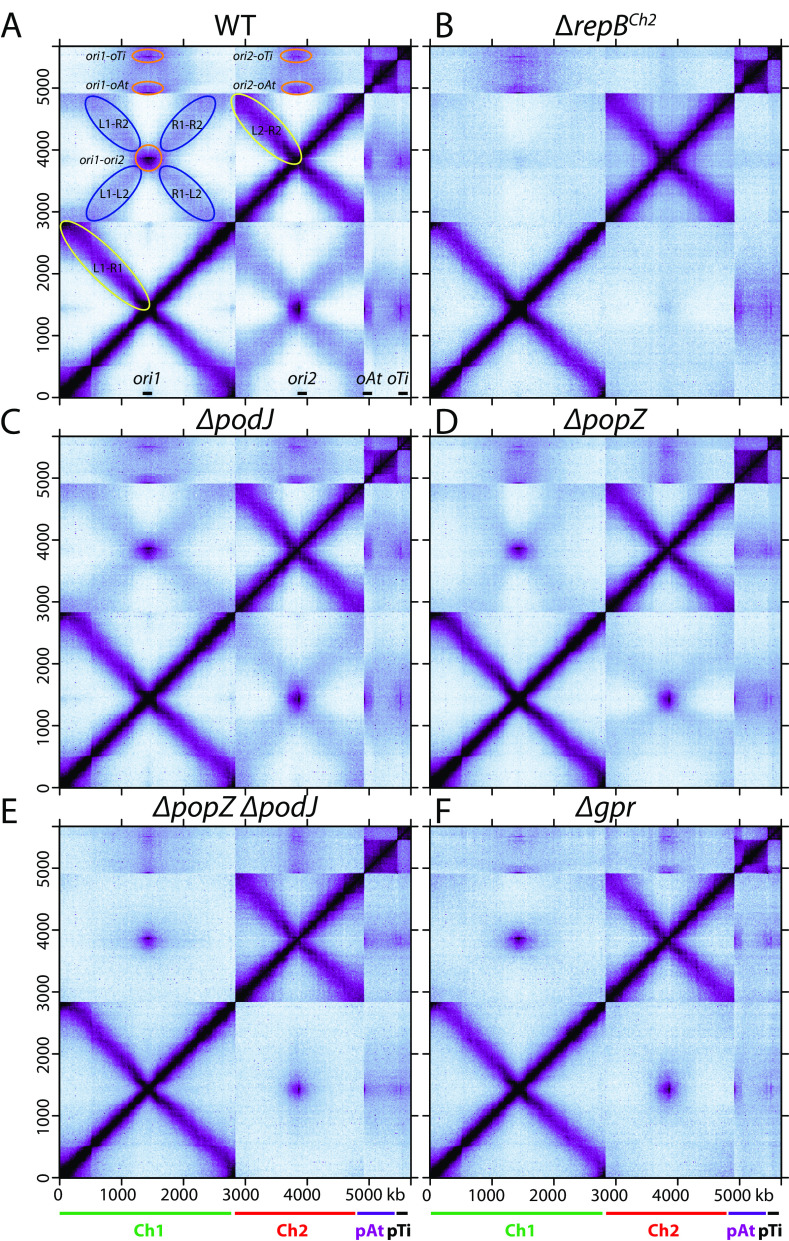
Genome-wide interactions of A. tumefaciens replicons. Normalized Hi-C contact maps displaying contact frequencies for pairs of 10-kb bins across the genome of A. tumefaciens in indicated strains. (A) Wild-type C58 (AtWX063) (11); (B) *ΔrepB^Ch2^* (AtWX089) (11); (C) *ΔpodJ* (AtWX283); (D) *ΔpopZ* (AtWX110); (E) *ΔpopZ ΔpodJ* (AtWX121); (F) *Δgpr* (AtWX286). *x* axis and *y* axis indicate genome positions. To better visualize contacts in the origin region of Ch1, the reference genome of Ch1 is rearranged with the origin (*ori1*) at the center and the two replication arms on either side. Ch1, Ch2, pAt, and pTi are indicated by green, red, purple, and black bars, respectively. In panel A, the positions of the four origins are indicated on the *x* axis. L1, R1, L2, and R2 indicate the left arm of Ch1, right arm of Ch1, left arm of Ch2, and right arm of Ch2, respectively. Interarm interactions on Ch1 and Ch2 are circled in yellow. Interactions between origins are circled in orange. The interactions between the arms of Ch1 and the arms of Ch2 are circled in blue. Quantitative analyses of interactions can be found in Fig. S4.

Next, to complement our single-cell microscopy experiments, we investigated how these polar organizers contribute to the genome-wide DNA organization using Hi-C. In the WT (11), within Ch1 and Ch2, there are intrachromosomal interactions between the chromosome arms, called interarm interactions (Fig. 3A, yellow ovals); between the replicons, there are two types of interreplicon interactions: the clustering of the origins (Fig. 3A, orange ovals) and the alignment of Ch1 and Ch2 in which the two chromosomes are aligned along the arms (Fig. 3A, X-shaped interactions indicated with blue ovals). Our previous study showed that in the absence of ParB1 or RepB^Ch2^ proteins, all the interchromosomal contacts between Ch1 and Ch2 were abolished (11) (Fig. 3B). Therefore, we used *ΔrepB^Ch2^* mutant as the background level of Ch1-Ch2 contacts. In *ΔpodJ*, *ΔpopZ*, and *Δgpr* single mutants, the most dramatic change was the reduction in the Ch1-Ch2 alignment interactions, which were reduced to 60.2%, 33.0%, and 13.7%, respectively (Fig. 3C, D, and F and Fig. S4A, B, and D). In the *ΔpopZ ΔpodJ* double mutant, Ch1-Ch2 alignment interactions were below background levels (Fig. 3E and Fig. S4A, B, and D). By comparison, the intrareplicon contacts were unaltered (Fig. 3C to F) and *ori1-ori2* interactions were only mildly affected (Fig. 3C to F and Fig. S4A to C). Similar results are obtained in a genetically and phylogenetically distinct wild-type strain, A. tumefaciens 15955, with substantially different gene content but similar genome architecture (11, 34) (Fig. S4E to G).

10.1128/mbio.00508-22.4 FIG S4 Quantification of interreplicon interactions. (A to D) Quantifications of *ori1-ori2* interactions (orange region) and Ch1-Ch2 alignment (blue regions) in different strains. (A and B) Regions used for quantification. Details can be found in Materials and Methods. Interactions in *ΔrepB^Ch2^* strain are set as the background (0%, black dotted lines). After subtracting background, the percentage of interactions relative to the WT (100%) is shown. (E to G) A. tumefaciens 15955 strain showed similar phenotype. Normalized Hi-C contact maps for 15955 WT (11) (E), 15955 *ΔpopZ* (IB173) (F), 15955 *ΔpodJ* (IB172) (G) grown in ATGN. Download FIG S4, PDF file, 2.5 MB. Copyright © 2022 Ren et al. 2022 Ren et al. https://creativecommons.org/licenses/by/4.0/ This content is distributed under the terms of the Creative Commons Attribution 4.0 International license.

Combining our microscopy (Fig. 2) and Hi-C results (Fig. 3), we postulated that the three polar organizers are required for the polar localization of the origins/centromeres, and this polar anchoring of the origins and the rod cell shape help linearly align Ch1 and Ch2, generating the X-shaped pattern in the Hi-C maps (Fig. 3A, blue ovals). However, our data showed that the polar organizers are not required for the *ori-ori* colocalization in the cell or their clustering interactions in Hi-C. These results raised the possibility that the ParB1 and RepB proteins directly interact to cluster together.

### ParB1 and RepB^Ch2^ interact when expressed in B. subtilis.

In our previous ChIP-seq experiments, we observed that ParB1 was not only enriched at *parS1* sites but also had an enrichment peak at *parS2* sites, albeit at much lower levels (Fig. S5A and C). Similarly, RepB^Ch2^ ChIP-seq showed strong enrichment at *parS2* sites but also weak enrichment at *parS1* sites (Fig. S5B and D). These ChIP-seq experiments were performed with formaldehyde cross-linking and could not distinguish whether the reciprocal enrichment was through direct ParB1-RepB^Ch2^ interactions or was indirectly bridged by other proteins in A. tumefaciens cells.

10.1128/mbio.00508-22.5 FIG S5 ChIP-seq enrichment of ParB1 and RepB^Ch2^ at cognate sites and reciprocal sites. (A) ParB1 enrichment in wild-type cells (11). Sequencing reads from ChIP and input samples were normalized to the total number of reads and plotted in 1-kb bins. *x* axis shows genome positions. (B) RepB^Ch2^ enrichment in wild-type cells (11). (C) High-resolution plots of ParB1 enrichment from panel A at 50-kb regions encompassing *ori1* (left) and *ori2* (right). *parS1* and *parS2* sites are indicated by blue and gray dotted lines, respectively. Data are plotted in 100-bp bins. (D) High-resolution plots of RepB^Ch2^ enrichment from panel B at 50-kb regions encompassing *ori1* (left) and *ori2* (right). Download FIG S5, PDF file, 0.6 MB. Copyright © 2022 Ren et al. 2022 Ren et al. https://creativecommons.org/licenses/by/4.0/ This content is distributed under the terms of the Creative Commons Attribution 4.0 International license.

**FIG 4:**
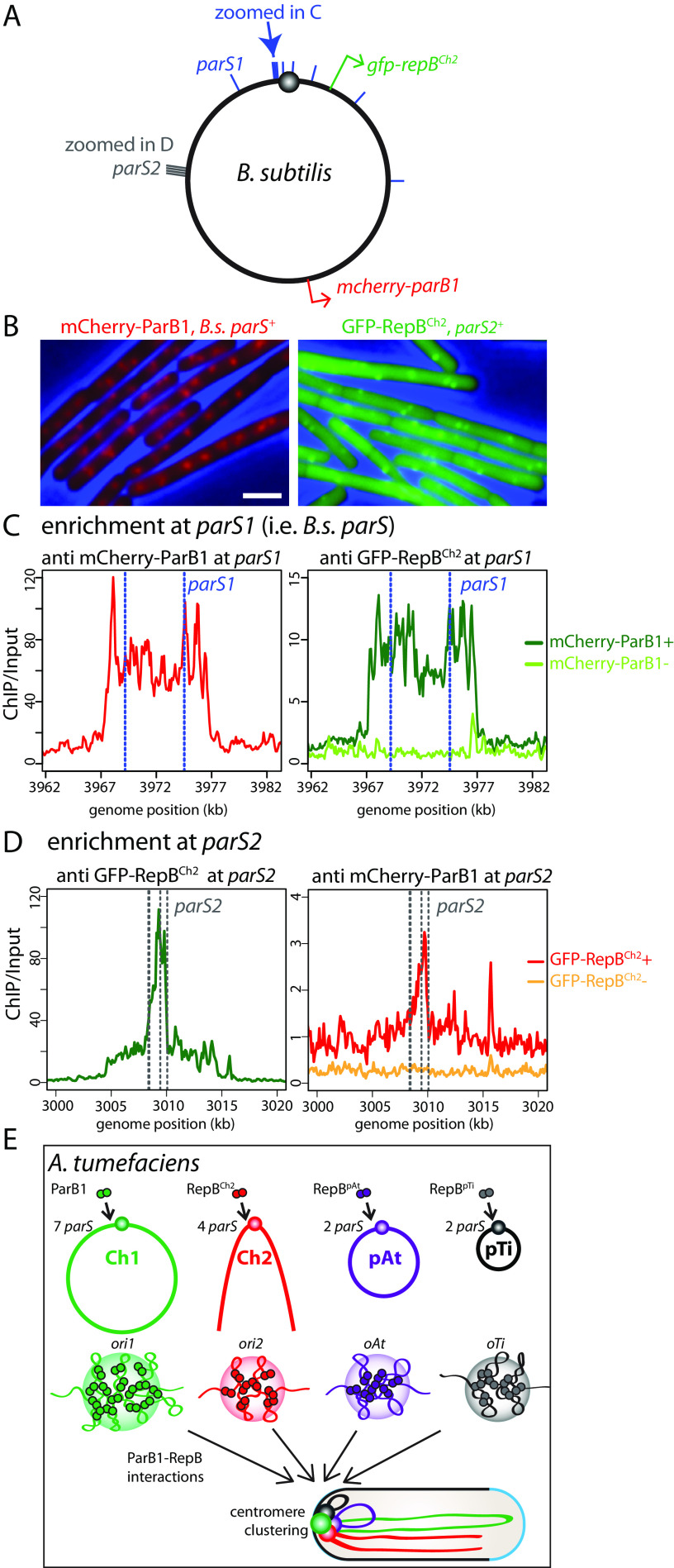
ParB1 and RepB^Ch2^ interact in B. subtilis. (A) Schematic of the B. subtilis chromosome. Blue bars indicate the position of the nine B. subtilis *parS* sites, which have the same consensus sequence as A. tumefaciens *parS1* sites. Blue arrow points to the two *parS1* sites shown in panel C. Gray bars indicate the cluster of *parS2* sites also shown in panel D. Red and green arrows indicate the locations from which *mcherry-parB1* and *gfp-repB^Ch2^* are expressed. (B) Expressing mCherry-ParB1 (left) and GFP-RepB^Ch2^ (right) in the presence of *parS1* and *parS2* leads to fluorescence focus formation in B. subtilis. (C, left) At *parS1* sites (blue dotted lines), mCherry-ParB1 had high enrichment. (Right) GFP-RepB^Ch2^ had low enrichment when mCherry-ParB1 was present, and no enrichment was seen when mCherry-ParB1 was absent. (D) At *parS2* sites (gray dotted lines), GFP-RepB^Ch2^ had high enrichment (left); mCherry-ParB1 has low enrichment when GFP-RepB^Ch2^ was present and no enrichment when GFP-RepB^Ch2^ was absent (right). (E) Schematic model of *ori* clustering. Centromeric ParB1 and RepB protein dimers bind to their *parS* sites (11) near the replication origins of the four replicons and spread to nearby regions to form nucleoprotein complexes. The three RepB nucleoprotein complexes interact with the ParB1 nucleoprotein complex, leading to the clustering of the four origins/centromeres.

To address whether the ParB1 and RepB^Ch2^ enrichment at the reciprocal sites required other A. tumefaciens factors, we set out to perform similar ChIP-seq experiments in a distantly related bacterium, B. subtilis. We expressed mCherry-ParB1 and GFP-RepB^Ch2^ in a B. subtilis strain lacking the native B. subtilis *parB* gene (Fig. 4A). The B. subtilis genome contains 9 B. subtilis *parS* sequences (Fig. 4A, blue bars), which have a consensus sequence identical to A. tumefaciens *parS1* (11, 34). For GFP-RepB^Ch2^ binding, we cloned the cluster of four A. tumefaciens *parS2* sequences from the A. tumefaciens *ori2* region and inserted it into the B. subtilis genome (Fig. 4A, gray bars). When expressed in B. subtilis cells, mCherry-ParB1 formed foci in the presence of *parS1*, similar to B. subtilis ParB localization (36); GFP-RepB^Ch2^ formed foci in the presence of *parS2*, similar to the localization of the chromosomal locus visualized using the *tetO*-TetR system (37, 38) (Fig. 4B). To investigate the genome-wide binding profiles of mCherry-ParB1 and GFP-RepB^Ch2^, we performed ChIP-seq experiments using anti-mCherry and anti-GFP antibodies. We found that when expressed singly, mCherry-ParB1 or GFP-RepB^Ch2^ was only enriched at the cognate *parS1* or *parS2* sites (Fig. 4C and D). However, when the fusions were coexpressed, ParB1 and RepB^Ch2^ were not only enriched at their cognate binding sites but also enriched at the reciprocal sites (Fig. 4C and D). Thus, ParB1 and RepB^Ch2^ are enriched to reciprocal sites when they are present together but in the absence of other A. tumefaciens proteins. Although it is possible that an unknown factor from B. subtilis is bridging ParB1-RepB^Ch2^ interactions, it is more likely that ParB1-RepB^Ch2^ directly interacts in B. subtilis.

We attempted to test ParB1 and RepB^Ch2^ interactions using bacterial two-hybrid assay (BACTH) (39) and *in vitro* pulldown assays, and we could not detect a positive ParB1-RepB^Ch2^ interaction (Fig. S6). There are two plausible explanations for why we could detect these interactions in the B. subtilis coexpression experiment but not using BACTH or *in vitro* pulldown experiments. First, *in vivo* spreading of ParB family proteins allows formation of a three-dimensional nucleoprotein complex (35, 36, 40, 41), possibly in a separated phase (42, 43). It is likely that the nucleation of ParB1/RepB^Ch2^ is important for ParB1-RepB^Ch2^ interactions, and our BACTH and *in vitro* pulldown experiments did not allow full nucleation of these proteins. Second, ParB1-RepB^Ch2^ interactions might be weak or transient, allowing detection in ChIP experiments with cross-linking *in vivo* but not in BACTH or *in vitro* pulldown.

10.1128/mbio.00508-22.6 FIG S6 ParB1-RepB^Ch2^ interactions could not be detected in BACTH or *in vitro* pulldown assays. (A) BACTH interactions between ParB1 and RepB^Ch2^. E. coli strain BTH101 (57) expresses protein fusions to different domains (T25 and T18) of an adenylate cyclase. T25 and T18 fused to the same leucine zipper domain (“zip”) from yeast GCN4 serve as both positive and negative controls (39). This experiment detected interactions between ParB1 and ParB1 and between RepB^Ch2^ and RepB^Ch2^ but not between ParB1 and RepB^Ch2^. (B) An SDS-PAGE gel of an *in vitro* pulldown experiment. Affinity-purified ParB1 polyclonal antibodies were crosslinked to magnetic protein A Sepharose beads, which were then incubated with 50 μg of ParB1 protein and 50 μg of RepB^Ch2^ proteins in 1 mL 1× PBS solution, singly or doubly. Similarly, beads crosslinked with purified RepB^Ch2^ antibodies were incubated with proteins. Proteins were eluted in sample loading buffer at 65°C and separated by stain-free precast 4 to 20% polyacrylamide gradient gels (4561096; Bio-Rad). L is for protein ladder (1610363; BioRad). The gels were imaged using ProteinSimple Fluorchem R gel documentation system. (C) An SDS-PAGE gel of an *in vitro* pulldown experiment using ParB1 beads similar to that in panel B. The beads were incubated with ParB1, RepB^Ch2^, 1 mM CTP, and 3 μM *parS1* and *parS2* DNA fragments. ParB1-RepB^Ch2^ interactions were not detected. (D) The SDS-PAGE gel in panel C was immunoblotted using RepB^Ch2^ antibodies. A small amount of RepB^Ch2^ protein could be detected when incubated with ParB1 beads alone, but this level did not increase in the presence of the ParB1 protein. Download FIG S6, PDF file, 1.3 MB. Copyright © 2022 Ren et al. 2022 Ren et al. https://creativecommons.org/licenses/by/4.0/ This content is distributed under the terms of the Creative Commons Attribution 4.0 International license.

## DISCUSSION

Many pathogens or symbionts across the bacterial kingdom contain multiple replicons, also known as a multipartite genome. It has been proposed that a multipartite genome gives these bacteria many competitive advantages (1, 2). For instance, splitting a genome into multiple replicons allows these species to duplicate their genomes faster and grow more rapidly; modulating replicon copy numbers allows for flexible gene dosage regulation and better adaption to new niches because individual secondary replicons often contain genes important for surviving a specific growth condition. Despite these advantages, maintaining a multipartite genome is challenging, especially for the nonessential replicons that are only beneficial in certain environments. An improved understanding of bacterial multipartite genome organization and interactions is emerging. Using A. tumefaciens as a model organism, we recently found that the four replicons interact with each other, and their replication origins/centromeres are clustered together (11). In this study, we identified the biological function for the clustering of origins. We found that disruption of origin clustering led to the loss of chromosome 2 and the plasmids. Thus, we establish that being clustered to the primary chromosome’s centromere is critical for the stable maintenance of the secondary replicons. We posit that origin clustering allows multiple replicons to be uniformly propagated and maintained, so that they can contribute when they are needed as conditions change. Although maintaining nonessential replicons poses a fitness burden for the cells in certain environments, this cost is balanced by the advantages they impart when conditions change and their gene content becomes more beneficial. Viewed in the context of a selfish genetic element, origin clustering is another mechanism by which a replicon such as a virulence plasmid can increase its own long-term stability and promote overall fitness of a facultative pathogen or symbiont, alternating between host association and free-living modes of growth (44).

We investigated the molecular mechanism of origin clustering. Since all the replicons have the origins localized to the cell pole, one possibility is that polar organizer proteins interact with each origin separately and indirectly cluster the origins together. In A. tumefaciens, three such proteins are known: PodJ, PopZ, and GPR (13–18, 20). We found that these polar organizers are not required for the colocalization or clustering of the origins, suggesting that ParB1-RepBs directly interact with each other and cluster together. Indeed, when we expressed mCherry-ParB1 and GFP-RepB^Ch2^ in the distantly related B. subtilis, we observed that ParB1 and RepB^Ch2^ not only enriched at their own binding sites but also enriched at reciprocal sites. Thus, our results indicate that ParB1 and RepB^Ch2^ interact with each other in the absence of other A. tumefaciens proteins and support the idea that centromeres intrinsically adhere to one another, leading to the *ori* clustering.

The absence of ParB1-RepB^Ch2^ interactions using purified components suggests that these interactions are weak and transient. Nevertheless, due to ParB1/RepB^Ch2^ spreading to nearby regions (35, 36, 40), such weak or transient interactions could be significant on chromatin in live cells and provide enough strength to tether the centromeres together. On the other hand, the adhesion between centromeres being weak and transient, as opposed to being strong and permanent, might be beneficial to the cells by allowing the separation of the centromeres when necessary. Indeed, the centromeres of the secondary replicons disassociate from the centromere of Ch1 just prior to their duplication (11). This dissociation enables the replicated centromeres to translocate to two opposite cell poles instead of being pulled to the same pole by *ori1*.

In a broader context, eukaryotic centromeres are usually heterochromatin and exist in compartments separated from the transcriptionally active compartments (45). A recent study showed that eukaryotic centromeres also tend to adhere to one another (46). Although the molecular mechanism of centromere clustering in eukaryotes is unknown, it is possible that this is also through direct interactions. We postulate that centromeric clustering is a conserved mechanism for maintaining multipartite genomes both in bacteria and in eukaryotes.

## MATERIALS AND METHODS

### General methods.

A. tumefaciens strains were derived from strain C58 (47) or 15955 (34). Cells were grown in defined minimal medium (33) (ATGN) or LB broth at 30°C with aeration. In liquid medium, when appropriate, antibiotics or supplements were added at the following concentrations: kanamycin (IB02120; IBI) at 150 μg/mL, carbenicillin (C-103-5; GoldBio) at 25 μg/mL, gentamicin (AC613980010; ACROS Organics) at 150 μg/mL, theophylline (T1633-100G; Sigma) at 2 mM, and AHL (N-3-oxooctanoyl-l-homoserine lactone) (O1764-10MG; Sigma) at 1 μM. Isopropyl-β-d-thiogalactopyranoside (IPTG) (DS102125; Dot Scientific) was added at 0.5 mM for expressing PodJ, PopZ, and GPR fusions or at 0.2 mM for expressing ParB1 and RepB^Ch2^ fusions from pWX970. Antibiotics were doubled when applied on solid medium. B. subtilis strains were derived from the prototrophic strain PY79 (48). Cells were grown in defined rich medium (CH) (49) at 37°C with aeration. Lists of strains, plasmids, oligonucleotides, and next-generation sequencing samples can be found in Table S1 in the supplemental material. Details of strain and plasmid construction can be found in Text S1.

10.1128/mbio.00508-22.7 TABLE S1 Strains, plasmids, oligonucleotides, next-generation sequencing samples used in this study. (A) Bacterial strains used in this study. (B) Plasmids used in this study. (C) Oligonucleotides used in this study. (D) Next-generation sequencing samples used in this study. Download Table S1, DOCX file, 0.1 MB. Copyright © 2022 Ren et al. 2022 Ren et al. https://creativecommons.org/licenses/by/4.0/ This content is distributed under the terms of the Creative Commons Attribution 4.0 International license.

10.1128/mbio.00508-22.8 TEXT S1 Supplemental Materials and Methods. Download Text S1, DOCX file, 0.1 MB. Copyright © 2022 Ren et al. 2022 Ren et al. https://creativecommons.org/licenses/by/4.0/ This content is distributed under the terms of the Creative Commons Attribution 4.0 International license.

### Hi-C.

The detailed Hi-C procedure for A. tumefaciens is the same as that previously described (11, 50). Briefly, exponentially growing cells were cross-linked with 3% formaldehyde at room temperature for 30 min and then quenched with 125 mM glycine. Cells were lysed using Ready-Lyse lysozyme (R1802M; Epicentre), followed by 0.5% SDS treatment. Solubilized chromatin was digested with HindIII for 2 h at 37°C. The digested ends were filled in with Klenow and Biotin-14-dATP, -dGTP, -dCTP, and -dTTP. The products were ligated in dilute reactions with T4 DNA ligase at 16°C for about 20 h. Crosslinks were reversed at 65°C overnight for about 20 h in the presence of EDTA, proteinase K, and 0.5% SDS. The DNA was then extracted twice with phenol-chloroform–isoamylalcohol (25:24:1) (PCI), precipitated with ethanol, and resuspended in 20 μL of 0.1× Tris-EDTA (TE) buffer. Biotin from nonligated ends was removed using T4 polymerase (4 h at 20°C) followed by extraction with PCI. The DNA was then sheared by sonication for 12 min with 20% amplitude using a Qsonica Q800R2 water bath sonicator. The sheared DNA was used for library preparation with the NEBNext UltraII kit (E7645) by following the manufacturer’s instructions for end repair, adapter ligation, and size selection. Biotinylated DNA fragments were purified using 10 μL streptavidin beads. DNA-bound beads were used for PCR in a 50-μL reaction mix for 14 cycles. PCR products were purified using Ampure beads (A63881; Beckman) and sequenced at the Indiana University Center for Genomics and Bioinformatics using a NextSeq 550.

Paired-end sequencing reads were mapped to the combined genome files of A. tumefaciens C58 (NCBI reference sequence GCA_000092025.1) or 15955 (NCBI reference sequence GCA_003666465.1) using the pipeline described previously (50, 51). The A. tumefaciens genome was divided into HindIII restriction fragments, and each read of a read pair was independently mapped onto its corresponding restriction fragment using Bowtie 2.1.0. Only read pairs for which both reads uniquely aligned to the genome were considered in subsequent steps. Based on where on the genome the two reads in the pair are mapped, read pairs were first classified as valid Hi-C products or as nonligation or self-ligation products (51). Only valid Hi-C products were considered below.

To create interaction matrices, the A. tumefaciens genome was divided into 10-kb bins. Valid Hi-C products were then assigned to individual bins (50, 51). Raw Hi-C contact maps can be biased due to the uneven distribution of restriction enzyme sites, differences in GC content, and the mappability of individual reads (51, 52). We therefore normalized raw contact maps using an iterative normalization procedure, implemented using the hiclib library for Python (https://github.com/mirnylab/hiclib-legacy) (51). Essentially, we converted the number of interactions, or read counts, into Hi-C scores by applying the following equation and iteratively repeating it for the resulting contact map after each cycle: mij = mij × (total reads)/(total reads in bin *i* × total reads in bin *j*) (51), in which mij represents the contact frequency value at row i and column j of the contact matrix. The iterative procedure was repeated until the maximum relative error of the total number of Hi-C scores in a bin was less than 10^−5^. Resulting matrices were normalized so that Hi-C scores for each row and column sum to 1. To put *ori1* at the center of Ch1, the reference genome of Ch1 starts at 1,400 kb. Subsequent analysis and visualization was done using R scripts that are available upon request.

### ChIP-seq.

Chromatin immunoprecipitation (ChIP) for A. tumefaciens and B. subtilis was performed similarly to a previously described protocol (11, 50). Briefly, cells were cross-linked using 3% formaldehyde for 30 min at room temperature and then quenched, washed, and lysed. Chromosomal DNA was sheared to an average size of 250 bp by sonication using a Qsonica Q800R2 water bath sonicator. The lysate was then incubated overnight at 4°C with appropriate antibodies. A volume of 4 μL of anti-ParB1 (11), anti-RepB^Ch2^ (11), or anti-GFP (53) antibodies was added to the lysates and rotated at 4°C overnight. The lysates then were incubated with magnetic protein A Sepharose (28951378; GE Healthcare/Cytiva) for 1 h at 4°C. After washes and elution, the immunoprecipitate was incubated at 65°C overnight to reverse the cross-links. The DNA was further treated with RNase A and proteinase K, extracted with PCI, resuspended in 50 μL 0.1× TE buffer, and used for library preparation with the NEBNext UltraII kit (E7645) and sequenced using the Illumina NextSeq550 platforms.

The sequencing reads were aligned to the combined A. tumefaciens C58 genome (NCBI no. GCA_000092025.1) or B. subtilis PY79 genome (NCBI reference sequence NC_022898.1) using CLC Genomics Workbench (CLC Bio, Qiagen). The mapped result was exported as a .csv file containing genome location and read number for each base pair in the genome. Exported data from ChIP and input samples were each normalized to the total number of reads, and then ChIP enrichment (ChIP/input) was calculated for each base pair. The results were plotted and analyzed using customized R scripts that are available upon request.

### WGS.

Exponentially growing cells were collected and frozen in liquid nitrogen for WGS. Qiagen DNeasy kit (69504) was used to extract genomic DNA (gDNA). gDNA was subjected to sonication for 12 min using 20% amplitude. Sonicated gDNA was used for library preparation with the NEBNext UltraII kit (E7645) and sequenced using the Illumina NextSeq550 platforms. The reads were mapped to the combined A. tumefaciens C58 genome (NCBI no. GCA_000092025.1) or B. subtilis PY79 genome (NCBI reference sequence NC_022898.1) using CLC Genomics Workbench (CLC Bio; Qiagen). The mapped result was exported as a .csv file containing genome location and read number for each base pair in the genome. Exported data from WGS samples were normalized to the total number of reads and then plotted using customized R scripts that are available upon request.

### Microscopy.

Fluorescence microscopy was performed on a Nikon Ti2E microscope equipped with a Plan Apo 100×/1.4 numeric aperture phase-contrast oil objective and a scientific complementary metal oxide semiconductor camera. Cells were immobilized using 2% agarose pads containing growth medium. Images were cropped and adjusted using MetaMorph software (Molecular Devices). Final figures were prepared in Adobe Illustrator.

### Image analysis.

Image analyses were performed using the MathWorks MATLAB-based program Oufti (54). Cell outlines were detected using the cellDetection module. Localizations of fluorescent foci were identified using the spotDetection module. Pre-Gaussian parameters were set as the following: wavelet scale = 0, low pass = 2, spot radius = 2, int. threshold = 0.4, min region size =4, fit radius = 2. Post-Gaussian parameters were minHeight = 0, minWidth =1.9, maxWidth = 10, Adjusted Squared Error = 0. The same parameters were used for all strains. After cellDection and spotDetection, manual inspection was employed to remove the cell meshes with wrongly detected cell outline or spots. The data were further analyzed and plotted in MATLAB.

### Immunoblot analysis.

Western blotting was performed similar to previously described (53). Samples in sample buffer (1610737; Bio-Rad) were heated for 5 min at 95°C and vortexed vigorously for 30 s before loading to denature the proteins and shear the genomic DNA. Proteins were separated by precast 4 to 20% polyacrylamide gradient gels (4561096; Bio-Rad) and electroblotted onto mini-polyvinylidene difluoride (PVDF) membranes using Bio-Rad Transblot Turbo system and reagents (1704156; Bio-Rad). The membranes were blocked in 5% nonfat milk in phosphate-buffered saline (PBS) with 0.5% Tween 20 and then probed with anti-ParB1 (1:10,000) (11) or anti-RepB^Ch2^ (1:10,000) (11) diluted into 3% bovine serum albumin (BSA) in 1× PBS–0.05% Tween 20. Primary antibodies were detected using Immun-Star horseradish peroxidase-conjugated goat anti-rabbit antibodies (1705046; Bio-Rad) and Western Lightning Plus ECL chemiluminescence reagents as described by the manufacturer (NEL1034001; Perkin Elmer). The signal was captured using the ProteinSimple Fluorchem R system.

### BACTH.

BACTH was carried out as previously described using Escherichia coli strain BTH101 (39). ParB1 and RepB^Ch2^ were fused to T18 and T25 of adenylate cyclase. If the proteins interact, these fusion proteins will generate a functional adenylate cyclase leading to the expression of *lacZ* and blue colonies on 5-bromo-4-chloro-3-indolyl-β-d-galactopyranoside (X-Gal) plates. Strains were grown to stationary phase in LB containing Amp at 50 μg/mL, Kan at 25 μg/mL, and IPTG at 500 μM; 5 μL of cells was spotted on LB agar plates containing Amp at 50 μg/mL, Kan at 25 μg/mL, IPTG at 500 μM, and X-Gal at 40 μg/mL. Plates were incubated at 30°C.

### Antibody purification and cross-linking to protein A beads.

A volume of 20 mL of anti-ParB1 or anti-RepB^Ch2^ serum (11) was purified using Affigel-15 (1536051; Bio-Rad), eluted in 100 mM glycine, pH 2.5, and dialyzed into 1× PBS as previously described (55). The purified antibodies then were cross-linked to 1 mL magnetic protein A Sepharose beads (28951378; GE Healthcare/Cytiva) by incubating antibodies and Sepharose beads in the presence of 5 mM disuccinimidyl suberate (Pierce) for 30 min (56). The reaction was quenched using 100 mM Tris, pH 7.5. The antibody-Sepharose beads were washed with 100 mM glycine, pH 2.5, and resuspended in 1 mL PBS. Detailed procedures of downstream pulldown experiments are described in the legends of Fig. S6B and C. A volume of 50 μL antibody-Sepharose beads was used for each assay.

### Quantification of the *ori1-ori2* interactions.

In Fig. S4A to D, interactions between the *ori1* (bin122 to bin163) and *ori2* (bin363 to bin402) regions were quantified. Hi-C interaction scores in the rectangular region shown in orange were summed up and multiple by two to count the mirrored region across the primary diagonal. The interactions in the wild-type C58 (Fig. 3B and Fig. S4A) were set to 100%. The interactions in *ΔrepB^Ch2^* (AtWX089) (11) (Fig. 3C and Fig. S4B) were set as the background (0%). Interactions in the mutants were calculated relative to the wild type after subtracting the background.

### Quantification of the interchromosomal interactions along the length.

In Fig. S4A to D, interchromosomal interactions along the chromosome arms were calculated by adding up the Hi-C interaction scores in the blue area and then multiplying by two (Fig. 3B and C and Fig. S4A and B). Specifically, the two overlapping parallelogram regions have their vertices located at (13, 285), (63, 285), (220, 492), (270, 492) and (13, 492), (63, 492), (220, 285), (270, 285) on the Hi-C matrices, respectively, but with the *ori1-ori2* interactions in the orange square region removed. The interactions in the wild-type C58 (Fig. 3B and Fig. S4A) were set at 100%. The interactions in *ΔrepB^Ch2^* (AtWX089) mutant (11) (Fig. 3C and Fig. S4B) were set as the background (0%). Interactions in the mutants were calculated relative to the wild type after subtracting the background.

### Data availability.

Hi-C, ChIP-seq, and whole-genome sequencing data were deposited in the Gene Expression Omnibus (accession no. GSE196319).
