# Supplementary figures and images for: Centromere Interactions Promote the Maintenance of the Multipartite Genome in Agrobacterium tumefaciens
Source: mBio. 2022 May 10;13(3):e00508-22. doi: 10.1128/mbio.00508-22 (PMC9239152; doi:10.1128/mbio.00508-22)

Ren Figure S1

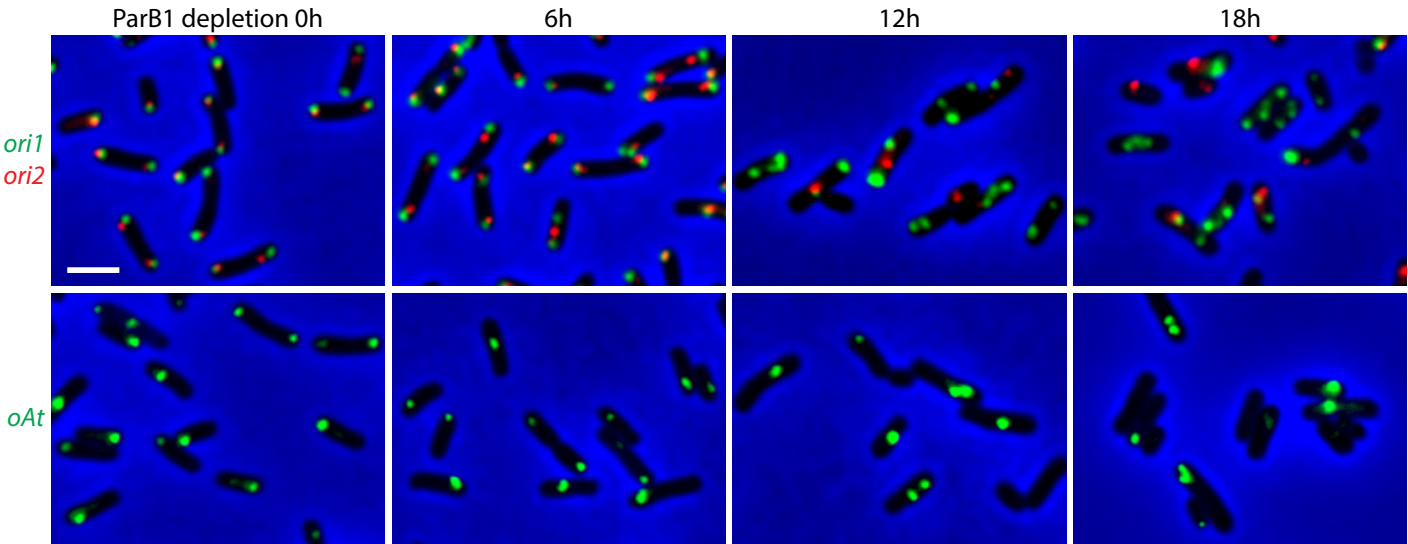

Supplement: FIG S1 [file mbio.00508-22-s0002.pdf]

Ren Figure S2

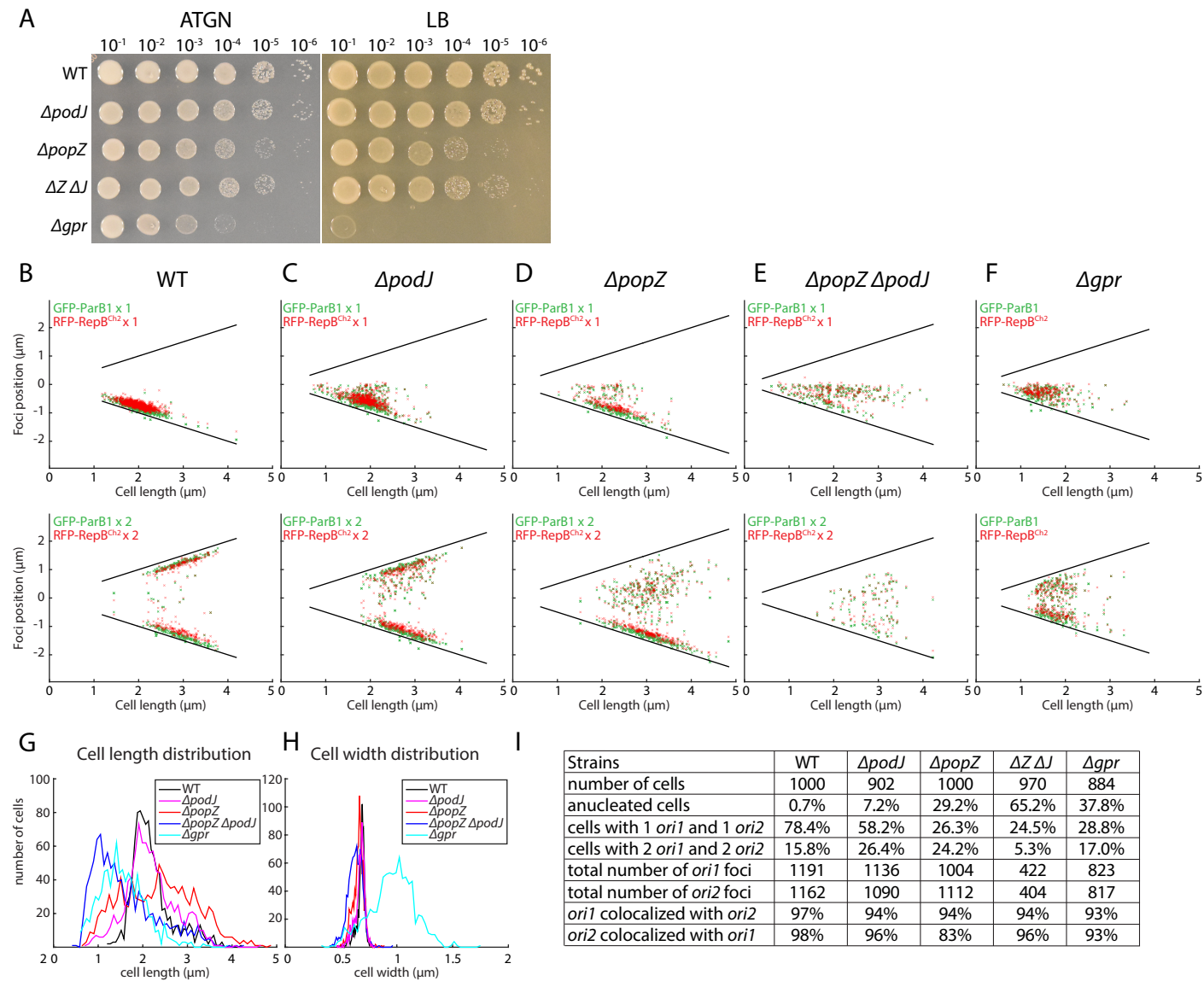

Supplement: FIG S2 [file mbio.00508-22-s0003.pdf]

Ren Figure S3

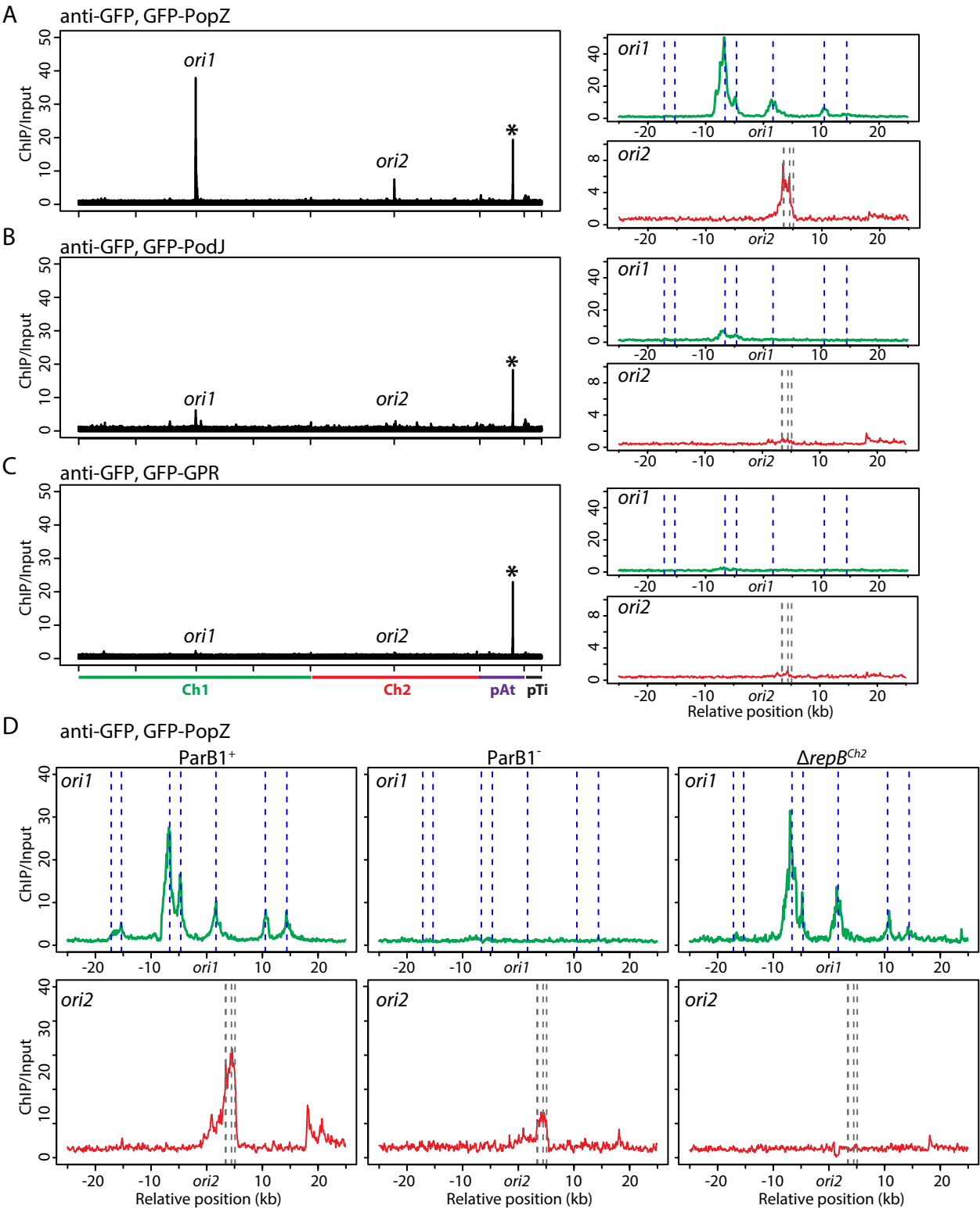

Supplement: FIG S3 [file mbio.00508-22-s0004.pdf]

Ren Figure S4

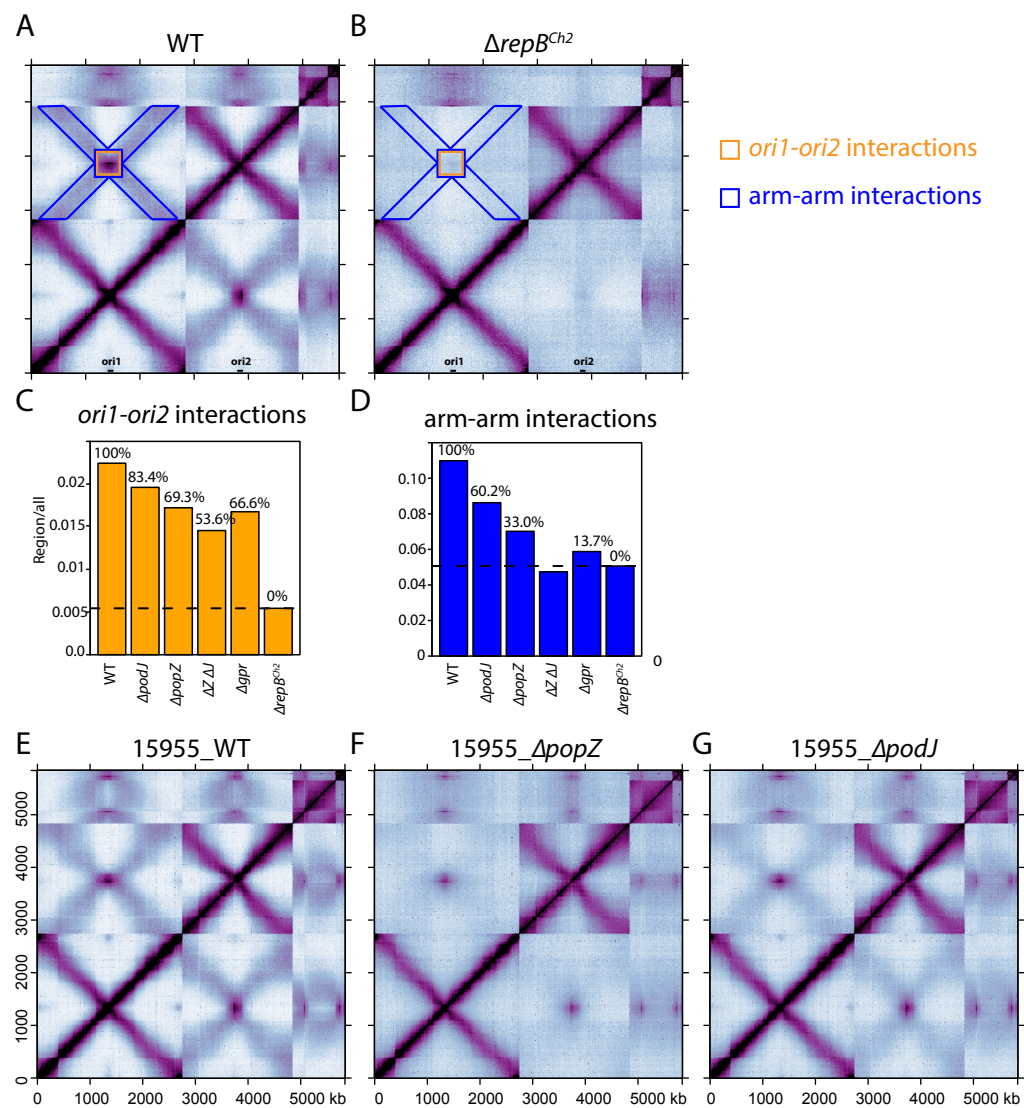

Supplement: FIG S4 [file mbio.00508-22-s0005.pdf]

Ren Figure S5

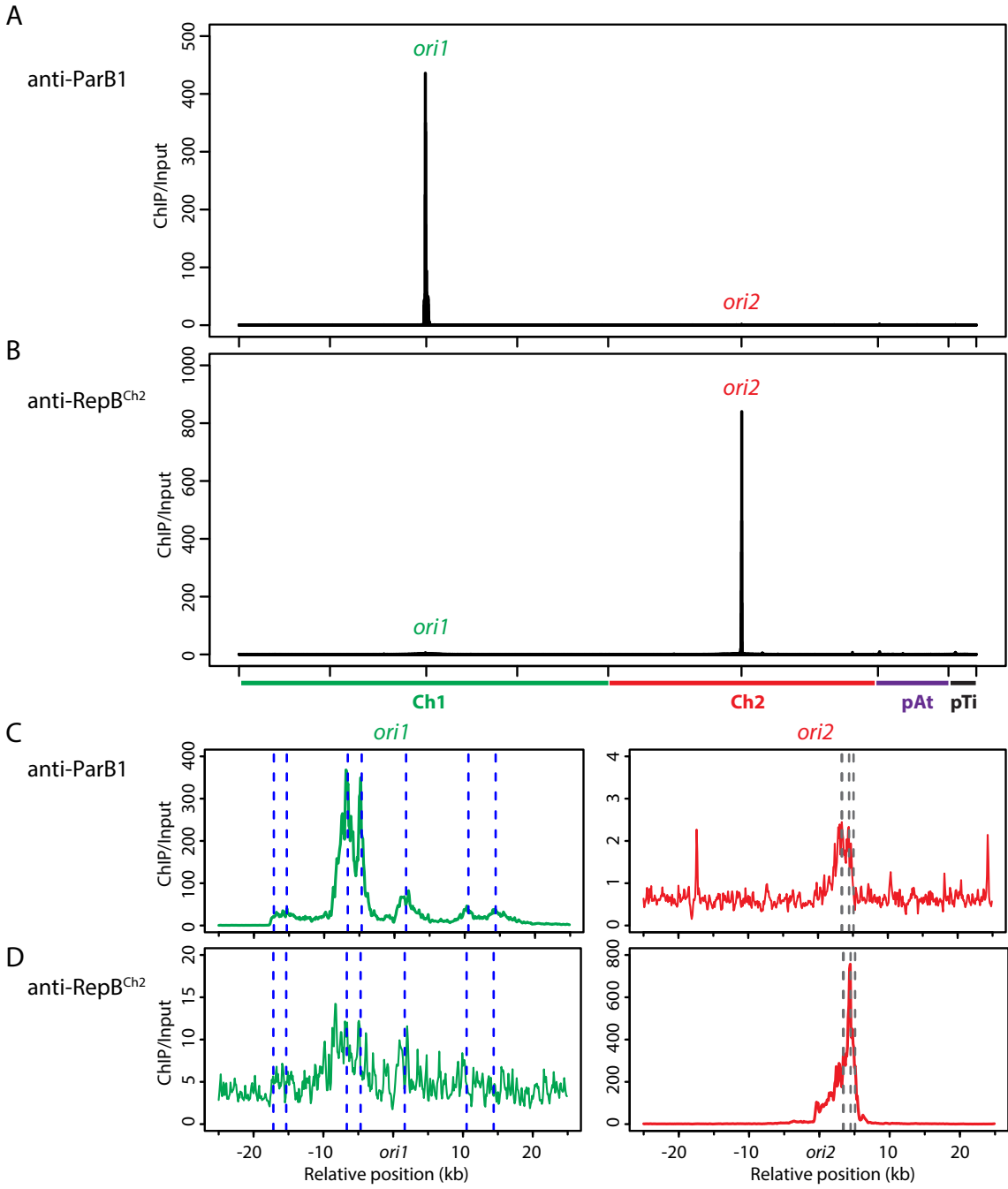

Supplement: FIG S5 [file mbio.00508-22-s0006.pdf]

Ren Figure S6

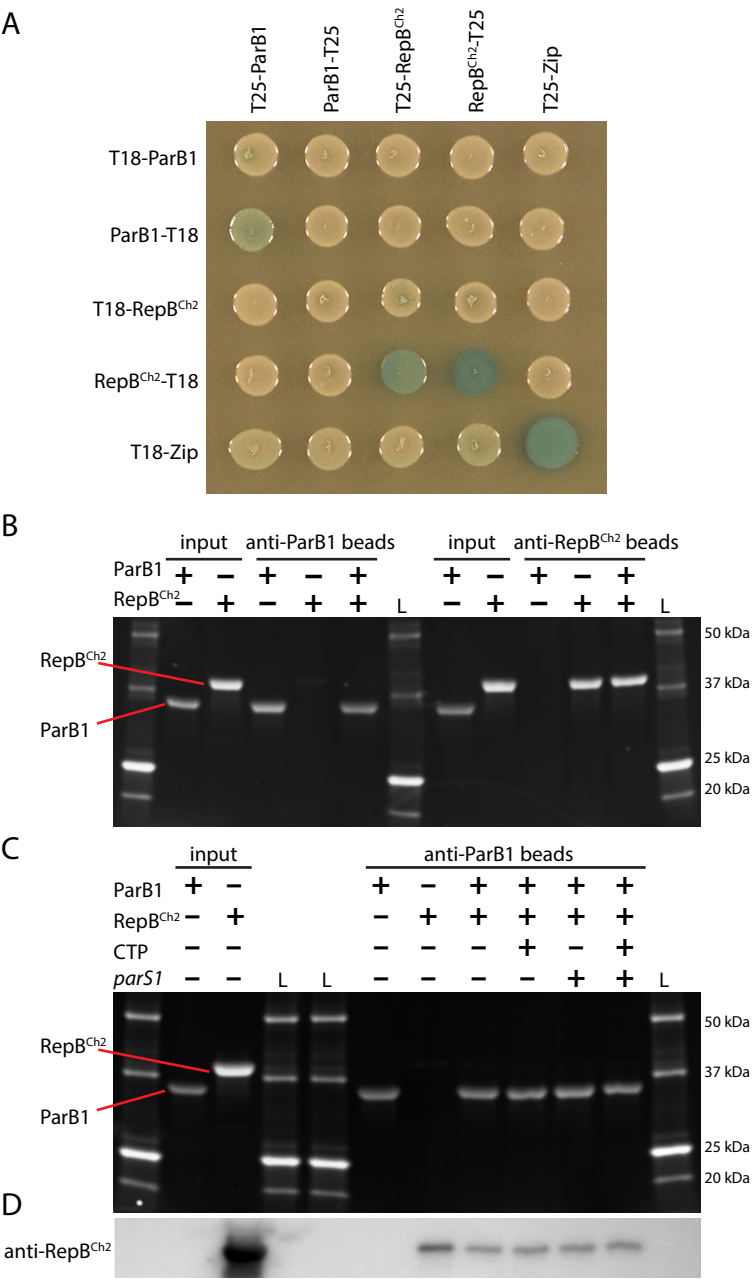

Supplement: FIG S6 [file mbio.00508-22-s0007.pdf]
