# Supplementary material for: Centromere Interactions Promote the Maintenance of the Multipartite Genome in Agrobacterium tumefaciens
Source: mBio. 2022 May 10;13(3):e00508-22. doi: 10.1128/mbio.00508-22 (PMC9239152; doi:10.1128/mbio.00508-22)
Supplement: TABLE S1 [file mbio.00508-22-s0001.docx]

**Table S1A. Bacterial strains used in this study.**

| **Strain** | **Genotype** | **Reference** | **Figure** |
| --- | --- | --- | --- |
| ***A. tumefaciens* used in main figures** | | | |
| AtWX063 | C58, wild type | (1) | 1B, 3A, S2A, S4ACD, S5ABCD |
| AtWX089 | C58, ∆*repB^Ch2^*(Atu3923/ATU_RS18280)::*amp* | (2) | 1B, 3B, S2A, S4BCD |
| AtWX192 | C58, ∆*traI*, *tetRA*::*gen* *PtraI-riboswitch-parB1*(Atu2828/ATU_RS13770) *traR* | (2) | 1B |
| AtWX356 | C58, *mcherry-parB^P1^-parS^P1^* inserted between Atu0047/ATU_RS00230 and Atu0048/ATU_RS00235, 50 kb from *ori1*, *ygfp-parB^pMT1^-par^pMT1^* inserted between Atu3973/ATU_RS18530 and Atu3974/ATU_RS18535, 57 kb from *ori2* | This study | 1CF |
| AtWX359 | C58, *ygfp-parB^pMT1^-parS^pMT1^* inserted between Atu5336/ATU_RS25500 and Atu5337/ATU_RS25505, 11 kb from *oAt* | (2) | 1CF |
| AtWX402 | C58, ∆*repB^Ch2^*(Atu3923/ATU_RS18280)::*amp*, *mcherry-parB^P1^-parS^P1^* inserted between Atu0047/ATU_RS00230 and Atu0048/ATU_RS00235, 50 kb from *ori1*, *ygfp-parB^pMT1^-par^pMT1^* inserted between Atu3973/ATU_RS18530 and Atu3974/ATU_RS18535, 57 kb from *ori2* | This study | 1DF |
| AtWX500 | C58, ∆*repB^Ch2^*(Atu3923/ATU_RS18280)::*amp*, *ygfp-parB^pMT1^-parS^pMT1^* inserted between Atu5336/ATU_RS25500 and Atu5337/ATU_RS25505, 11 kb from *oAt* | This study | 1DF |
| AtWX496 | C58, ∆*traI*, *tetRA*::*gen* *PtraI-riboswitch-parB1*(Atu2828/ATU_RS13770) *traR*,  *mcherry-parB^P1^-parS^P1^* inserted between Atu0047/ATU_RS00230 and Atu0048/ATU_RS00235, 50 kb from *ori1, ygfp-parB^pMT1^-par^pMT1^* inserted between Atu3973/ATU_RS18530 and Atu3974/ATU_RS18535, 57 kb from *ori2* | This study | 1EF, S1 |
| AtWX498 | C58, ∆*traI*, *tetRA*::*gen* *PtraI-riboswitch-parB1*(Atu2828/ATU_RS13770) *traR*, *ygfp-parB^pMT1^-parS^pMT1^* inserted between Atu5336/ATU_RS25500 and Atu5337/ATU_RS25505, 11 kb from *oAt* | This study | 1EF, S1 |
| AtWX277 | C58, *ygfp-parB^pMT1^-parS^pMT1^* inserted between Atu0047/ATU_RS00230 and Atu0048/ATU_RS00235, 50 kb from *ori1* | (2) | 1F |
| AtWX295 | C58, *ygfp-parB^pMT1^-parS^pMT1^* inserted between Atu3973/ATU_RS18530 and Atu3974/ATU_RS18535, 57 kb from *ori2* | (2) | 1F |
| AtWX351 | C58, *ygfpP-parB^pMT1^-parS^pMT1^* inserted between Atu6047/ATU_RS23235 and Atu6048/ATU_RS23240, 4 kb from *oTi* | (2) | 1F |
| AtWX263 | C58*,* carrying pWX970*,* pSRKKm *Plac rfp-repB^Ch2^* (Atu3923/ATU_RS18280) *terminator Plac egfp-parB1* (Atu2828/ATU_RS13770) *terminators* | (2) | 2A, S2B |
| AtWX307 | C58, ∆*podJ* (Atu0499/ATU_RS02460), containing pWX970 | This study | 2B, S2C |
| AtWX303 | C58*,* ∆*popZ* (Atu1720/ATU_RS08420)*,* containing pWX970 | This study | 2C, S2D |
| AtWX305 | C58, ∆*popZ* (Atu1720/ATU_RS08420) ∆*podJ* (Atu0499/ATU_RS02460), containing pWX970 | This study | 2D, S2E |
| AtWX309 | C58, ∆*gpr* (Atu1348/ATU_RS06650), containing pWX970 | This study | 2E, S2F |
| AtWX283 | C58, ∆*podJ* (Atu0499/ATU_RS02460) | This study | 3C, S2A, S4CD |
| AtWX110 | C58, ∆*popZ* (Atu1720/ATU_RS08420) | This study | 3D, S2A, S4CD |
| AtWX121 | C58, ∆*popZ* (Atu1720/ATU_RS08420) ∆*podJ* (Atu0499/ATU_RS02460) | This study | 3E, S2A, S4CD |
| AtWX286 | C58*, ∆gpr* (Atu1348/ATU_RS06650) | This study | 3F, S2A, S4CD |
| ***A. tumefaciens* used for strain building and in supplemental figures** | | | |
| AtWX234 | C58, containing pWX822, pSRKKm *msfgfp-popZ* (Atu1720/ATU_RS08420) | This study | S3A |
| AtWX265 | C58, containing pMAT3, pSRKKm *msfgfp*-*podJ* (Atu0499/ATU_RS02460) | This study | S3B |
| AtWX236 | C58, containing pJZ253, pSRKGm *gfp-gpr* (Atu1348/ATU_RS06650) | This study | S3C |
| AtWX289 | C58, ∆*traI*, *tetRA*::*gen* *PtraI-riboswitch-parB1*(Atu2828/ATU_RS13770) *traR*, pSRKKm *msfgfp-popZ* (Atu1720/ATU_RS08420) | This study | S3D |
| AtWX291 | C58, ∆*repB^Ch2^*, pSRKKm *msfgfp-popZ* (Atu1720/ATU_RS08420) | This study | S3D |
| AtWX486 | C58, ∆*traI*, *tetRA*::*gen* *PtraI-riboswitch-parB1*(Atu2828/ATU_RS13770) *traR*, *ygfp-parB^P1^-parS^P1^* inserted between Atu0047/ATU_RS00230 and Atu0048/ATU_RS00235, 50 kb from *ori1* | This study |  |
| AtWX050 | 15955, wild type | (3) | S4E |
| IB172 | 15955, ∆*podJ* (ISGA_411) | This study | S4F |
| IB173 | 15955, ∆*popZ* (ISGA_1749) | This study | S4G |
| ***B. subtilis* strains used in main figures** | | | |
| BWX5333 | *pelB::Psoj* *mcherry-parB1_At_* *tet, parS2_At_* cluster at -91° *kan, ∆parB_Bs_ spec* | This study | 4BCD |
| BWX5359 | *ycgO::Phyperspank-optRBS-mgfpmut3-repB^Ch2^*_At_ *cat*, *parS2_At_* cluster at -91° *kan, ∆parB_Bs_ spec* | This study | 4BCD |
| BWX5341 | *pelB::Psoj* *mCherry-parB1_At_* *tet*, *ycgO::Phyperspank-optRBS-mgfpmut3-repB^Ch2^*_At_ *cat*, *∆parB_Bs_ spec* | This study | 4C |
| BWX5353 | *pelB::Psoj* *mCherry-parB1_At_* *tet*, *ycgO::Phyperspank-optRBS-mgfpmut3-repB^Ch2^*_At_ *cat*, *parS2_At_* cluster at -91° *kan, parS∆9, ∆parB_Bs_ (∆parS) spec* | This study | 4D |
| ***B. subtilis* strains used for strain building and in supplemental figures** | | | |
| AG1468 | *∆spo0J::spec, trpC2, pheA1* | (4) |  |
| BWX2423 | *∆parB (∆parS) spec* | (5) |  |
| BWX3212 | *parS*∆9 no a.b. | (6) |  |
| BWX3379 | *parS*∆9 no a.b., *parS* at -91˚ *ytuF* *kan* | (7) |  |
| BWX5258 | *pelB::Psoj* *mcherry-parB1_At_* *tet* | This study |  |
| BWX5260 | *ycgO::Psoj mgfpmut3-repB^Ch2^_At_ cat* | This study |  |
| BWX5265 | *parS2_At_* cluster at -91° *kan* | This study |  |
| BWX5309 | *parS∆9 no a.b., ycgO::Phyperspank-optRBS-mgfpmut3- repB^Ch2^_At_ cat* | This study |  |
| BWX5329 | *ycgO::Phyperspank* *mgfpmut3-repB^Ch2^_At_* *cat*, *parS∆9* | This study |  |
| BWX5349 | *pelB::Psoj* *mCherry-parB1_At_* *tet*, *ycgO::Phyperspank-optRBS-mgfpmut3-repB^Ch2^_At_* *cat*, *parS2_At_* cluster at -91° *kan, ∆parB_Bs_ spec* | This study |  |

**Table S1B. Plasmids used in this study.**

| **Plasmid** | **Description** | **Reference** |
| --- | --- | --- |
| pFHC2973 | The plasmid carries *cfp-parB^P1^* and *ygfp-parB^pMT1^* | (8) |
| pGM9 | pNPTS138 *∆podJ* (Atu0499/ATU_RS02460) (*kan*) | Fuqua Lab, unpublished |
| pIB315 | pNPTS138 15955 ∆*popZ* (ISGA_1749) (*kan*) | This study |
| pIB316 | pNPTS138 15955 ∆*podJ* (ISGA_411) (*kan*) | This study |
| pJW005 | *yhdG::Phyperspank-opt.rbs-sirA (phleo)* | (9) |
| pJZ253 | pSRKGm *Plac gfp-gpr* (Atu1348/ATU_RS06650) (*kan*) | (10) |
| pJZ298 | pBSKII+ plasmid with *sacB* carb carrying 2kb sequencing homologous to *gpr* (Atu1348/ATU_RS06650) | (10) |
| pKNT18 | BACTH plasmid contains MCS *t18* (*amp*) | (11) |
| pKNT25 | BACTH plasmid contains MCS *t25* (*kan*) | (11) |
| pKT18 | BACTH plasmid contains *t18* MCS (*amp*) | (11) |
| pKT25 | BACTH plasmid contains *t25* MCS (*kan*) | (11) |
| pKT25zip | BACTH Plasmid was used to express *t25-zip* (*kan*) | (11) |
| pUT18Czip | BACTH Plasmid was used to express *t18-zip* (*amp*) | (11) |
| pMAT3 | pSRKKm *Plac msfgfp-podJ* (Atu0499/ATU_RS02460) (*kan*) | Fuqua Lab, unpublished |
| pNPTS138 | *oriT sacB kan* | (12) |
| pSRKKm | Broad host-range, *Plac* (*kan*) | (13) |
| pSRKKm msfGFP | pSRKKm *Plac msfgfp* (*kan*) | (14) |
| mini-Tn7 | pUC18-mini-Tn7T *gen Plac ha* | (14) |
| pWX294 | pACYC origin with MCS (*amp*) | This study |
| pWX563 | *pelB::Psoj-mgfpmut3-spo0J (parS*)* (*tet*) | (5) |
| pWX564 | *pelB::Psoj-mcherry-spo0J (parS*) (tet)* | (15) |
| pWX588 | *ycgO::Pspank* (optRBS) gfp-spo0J (parS*) cat* | This study |
| pWX822 | pSRKKm *Plac* *msfgfp-popZ* (Atu1720/ATU_RS08420) (*kan*) | (13) |
| pWX839 | pNPTS138 ∆*popZ* (Atu1720/ATU_RS08420) (*kan*) | This study |
| pWX845 | BACTH Plasmid was used to express *t25-parB1* (kan) | This study |
| pWX846 | BACTH Plasmid was used to express *t25-repB^Ch2^* (*kan*) | This study |
| pWX847 | BACTH Plasmid was used to express *parB1-t25* (*kan*) | This study |
| pWX848 | BACTH Plasmid was used to express *repB^Ch2^-t25* (*kan*) | This study |
| pWX849 | BACTH Plasmid was used to express *t18-parB1* (*amp*) | This study |
| pWX850 | BACTH Plasmid was used to express *t18-repB^Ch2^* (*amp*) | This study |
| pWX851 | BACTH Plasmid was used to express *parB1-t18* (*amp*) | This study |
| pWX852 | BACTH Plasmid was used to express *repB^Ch2^-t18* (*amp*) | This study |
| pWX854 | pNPTS138 *repB^Ch2^* (Atu3923/ATU_RS18280)::*ampR* (*kan*) | This study |
| pWX915 | pACYC *terminator* *Ppen* (*amp*) | This study |
| pWX916 | pACYC *terminator* *Ppen* *cfp-parB^P1^-parS^P1^* (*amp*) | This study |
| pWX930 | pNPTS138 *Ppen* *cfp-parB^P1^-parS^P1^* kan at Atu3054/ATU_RS14060 | This study |
| pWX936 | pNPTS138 PT7strong *cfp-parB^P1^-parS^P1^* at Atu3054/ATU_RS14060 | This study |
| pWX962 | pNPTS138 PT7strong *cfp-parB^P1^-parS^P1^* at Atu0048/ATU_RS00235 | This study |
| pWX967 | pNPTS138 PT7strong *yGFP-parB^pMT1^-parS^pMT1^* at Atu3973/ATU_RS18530 | This study |
| pWX970 | pSRKKm *Plac rfp-repB^Ch2^* (Atu3923/ATU_RS18280) *terminator* (Atu2828/ATU_RS13770) *parB1-egfp Plac terminators* | (2) |
| pWX995 | pNPTS138 terminators PT7strong *mcherry-parB^P1^-parS^P1^* at Atu0048/ATU_RS00235 | This study |
| pWX1005 | pNPTS138 *yGFP-parB^pMT1^-parS^pMT1^* Atu5337/ATU_RS25505 | (2) |

**Table S1C. Oligonucleotides used in this study.**

| **Oligo** | **Sequence** | **Use** |
| --- | --- | --- |
| oML83 | cctcatcctcttcatcctc | sequencing |
| oML85 | AATAGCGTCCTTGCTCTCGT | sequencing |
| IBE140 | ggatccagtagcctcgatcatgtcgggg | IB172 |
| IBE141 | catccgttgcgaaacggttacatcttctcgctcgcttcgc | IB172 |
| IBE142 | gcgaagcgagcgagaagatgtaaccgtttcgcaacggatg | IB172 |
| IBE143 | gctagcagccagcgttccgcgccggaaa | IB172 |
| IBE144 | ttcagcgggaaaagccgctc | IB172 |
| IBE145 | cgtagcgcccacgagaccgc | IB172 |
| IBE146 | ggatccactggtcgtcgttgtcggata | IB173 |
| IBE147 | atgcggacgaacagagcctacatatcaatccccggtttcc | IB173 |
| IBE148 | ggaaaccggggattgatatgtaggctctgttcgtccgcat | IB173 |
| IBE149 | gctagcactgctgttccatcagcttgc | IB173 |
| IBE150 | cagaccttgtccacgaaggc | IB173 |
| IBE151 | tcgatgaagtgccggcggca | IB173 |
| oWX439 | TCCTTCTGCTCCCTCGCTCAG | BWX5265 |
| oWX776 | atgggctgggaagccagcagcgag | sequencing |
| oWX998 | AAACCCGGGacataaggaggaactactatgagtaaagg | pWX588 |
| oWX999 | tttGCTAGCcagagtggaggcaagaacgccttaaccc | pWX588 |
| oWX1279 | CTAATCCGACAGCTAACCTCGTAGGCG | BWX5265 |
| oWX1282 | CGATAAAGTCGGACCAGGGATGCTCGG | BWX5265 |
| oWX1283 | TCCTATTTTCAGGCAGTGACGCCG | sequencing |
| oWX1782 | TGAGTTAGCTCACTCATTAGGC | sequencing |
| oWX1783 | ACCAGGCGGAACATCAATGTGG | sequencing |
| oWX1789 | CATCTGTCCAACTTCCGCGAC | sequencing |
| oWX1790 | CCTCTTCGCTATTACGCCAGC | sequencing |
| oWX1835 | GCCAGGGTTTTCCCAGTCACGA | sequencing |
| oWX1854 | CGCCAGGGTTTTCCCAGTCACGAC | sequencing |
| oWX1855 | TCACACAGGAAACAGCTATGAC | sequencing |
| oWX2044 | caatttcacacaggaaacagcatATGAGTAAAGGTGAAGAACTGTTCACC | pWX822 |
| oWX2046 | ggatccTCCAGATCCTTTGTATAGTTCATCCATGCCGTG | pWX822 |
| oWX2051 | TATACAAAGGATCTGGAggatccgctcagccaagtgtcgcgcgtgaac | pWX822 |
| oWX2052 | ctcgaggtcgacggtatcgataagcttttagcggcgcgagccgcgcgccacacg | pWX822 |
| oWX2060 | ggaaagcgggcagtgagcgc | sequencing |
| oWX2061 | GAAGGTTATGTACAGGAGCGCACC | sequencing |
| oWX2076 | tggcgccaagcttctctgcaggatatgacacagagtgccgatttaag | sequencing |
| oWX2077 | gctagcgaattcgtggatccagatctaacccgccatgcccacctcc | sequencing |
| oWX2160 | CTGGCGCCAAGCTTCTCTGCAGGATtgccaaggcaactgtctatcg | pWX839, sequencing |
| oWX2161 | ggcagcgattgtagcctgcggaatatcaatccccggtttctactc | pWX839 |
| oWX2162 | gagtagaaaccggggattgatattccgcaggctacaatcgctgcc | pWX839, sequencing |
| oWX2163 | AGCTAGCGAATTCGTGGATCCAGATagctggttttccatcagcttgc | pWX839, sequencing |
| oWX2190 | cgctctagagTAACACACAGGAAACAGCTatgagtgatgatctttcgaagcg | pWX847, pWX851 |
| oWX2191 | atgcccgggcCGAACCGCTACCtttctgctccagcagcc | pWX847, pWX851 |
| oWX2193 | atgCCCGGGttatttctgctccagcagcc | pWX845, pWX849 |
| oWX2194 | cgctctagagTAACACACAGGAAACAGCTatgagcagaaaacagatattcgc | pWX848, pWX852 |
| oWX2195 | atgcccgggcCGAACCGCTACCctgcttggaccggtattcg | pWX848, pWX852 |
| oWX2197 | atgCCCGGGttactgcttggaccggtattcg | pWX846, pWX850 |
| oWX2202 | cgctctagagGGCAGCGGTagtgatgatctttcgaagcg | pWX845, pWX849 |
| oWX2203 | cgctctagagGGCAGCGGTagcagaaaacagatattcgc | pWX846, pWX850 |
| oWX2291 | ggctgattggcatgacaatatttgacgtgcg | sequencing |
| oWX2292 | gttctgcgatcggcagatagacagtcacgg | sequencing |
| oWX2377 | GGCTTCCTTTGTTATCAAGCGCAG | sequencing |
| oWX2385 | gctGAATTCCCCGCGAAAGCGGGGTTTTTTTTTCCGGTGGAAACGAGGTCATCATTTC | pWX915 |
| oWX2386 | tttaagcttGAATATTTGATTGATCGTAACCAGATGAAGC | pWX915 |
| oWX2387 | TCAATCAAATATTCaagctTaaaggAggtggaaacATGAGTAAAGGAGAAGAACTTTTC | pWX916 |
| oWX2388 | TCTTAAATGACTCGCGAGAACTCGAGTTAATAGTGAAATTTGAATGGCGAAAG | pWX916 |
| oWX2389 | CTTTCGCCATTCAAATTTCACTATTAACTCGAGTTCTCGCGAGTCATTTAAGACCG | pWX916 |
| oWX2390 | GCCGataCTGCAGatGTCGACatGGATCCgtgaaatcgtggcgatttcaccttg | pWX916 |
| oWX2395 | TCTTCGCTATTACGCCAGATCC | sequencing |
| oWX2396 | CCGTCAATTGTCTGATTCGTTACC | sequencing |
| oWX2397 | GATGACGGTAACTACAAAACCC | sequencing |
| oWX2407 | CTCTAGAtagcGCATGCtGAATTC | pWX930, pWX962 |
| oWX2408 | GGTTATGCTAGTTATTGCTCAGCC | pWX930, pWX962 |
| oWX2420 | GGCGCCAAGCTTCTCTGCAGGATatccagttacgtgctggcggcaggatc | pWX930 |
| oWX2421 | GAATTCaGCATGCgctaTCTAGAGctattttgggatagctcgaaccgtg | pWX930 |
| oWX2422 | GGCTGAGCAATAACTAGCATAACCcggcaggcatatgaaaccggattg | pWX930 |
| oWX2423 | CTAGCGAATTCGTGGATCCAGATatctgccatgtggaacgatggtgaggg | pWX930 |
| oWX2424 | ttctgccgctccgatcaaaacagg | sequencing |
| oWX2425 | ccggactccacatccgcagatttc | sequencing |
| oWX2426 | GGCCTTCTGCTTAGCTAGAGCGGC | sequencing |
| oWX2431 | TAATACGACTCACTATAGGGAGACCACAACGCTTCATCTGGTTACGATCAATC | pWX936 |
| oWX2432 | GGTCTCCCTATAGTGAGTCGTATTAATTTCGAAATGATGACCTCGTTTCCACC | pWX936 |
| oWX2497 | AAGTCAAGTTTGAAGGTGATACCC | sequencing |
| oWX2502 | CGCCAAGCTTCTCTGCAGGATATCgaacgtcgatattggcctcgaatg | pWX962, sequencing |
| oWX2503 | AATTCaGCATGCgctaTCTAGAGtcagcccgcctgctttgctttcag | pWX962 |
| oWX2504 | GGCTGAGCAATAACTAGCATAACCagcggccgcctggtatttccag | pWX962 |
| oWX2505 | TAGCGAATTCGTGGATCCAGATATCtgcgggttatgcgctgccggcc | pWX962, sequencing |
| oWX2506 | acaatattggcctgatggaggacc | sequencing |
| oWX2507 | aaaagggacaggacacgctgttcc | sequencing |
| oWX2508 | CGCCAAGCTTCTCTGCAGGATATCaacgcgcgaaaaactgttgacg | sequencing |
| oWX2511 | AGCGAATTCGTGGATCCAGATATCgacactggaggatatgggaacattc | sequencing |
| oWX2530 | cggagaaggtcatgtcgagcg | sequencing |
| oWX2530 | cggagaaggtcatgtcgagcg | sequencing |
| oWX2531 | aaggcgttcagcgtgaaactcagg | sequencing |
| oWX2560 | catgagctcgagGCCTGATCCGCCAGATCCttacttaactgcgtctttcagtgcc | sequencing |
| oWX2563 | ccgaatcgtatagagattcttcg | BWX5258 |
| oWX2564 | ggactcgagatgagtgatgatctttcgaagcg | BWX5258 |
| oWX2566 | GGACTCGAGatgagcagaaaacagatattcgc | BWX5260 |
| oWX2567 | ATGGGATCCtcactgcttggaccggtattcgg | BWX5260 |
| oWX2568 | TTTAAAGGATTTGAGCGTAGCG | sequencing |
| oWX2569 | atgtgattcttccacaatgcctcgagtcaccctgtaaacacttcgccatc | BWX5265 |
| oWX2570 | gcgaagtgtttacagggtgactcgaggcattgtggaagaatcacatttgc | BWX5265 |
| oWX2571 | CGAACGGTACTGAGCGAGGGAGCAGAAGGAgtaatcggtatcttgcaagtatcc | BWX5265 |
| oWX2584 | TTCaagctTaaaggAggtggaaacatggtcagcaagggagaggaag | pWX995 |
| oWX2585 | CTGCTCGACcaTGAGCTCGAATTCtttgtataattcgtccattccacc | pWX995 |
| oWX2589 | cttcctctcccttgctgaccatgtttccaccTcctttAagcttG | pWX995 |
| oWX2590 | ggtggaatggacgaattatacaaaGAATTCGAGCTCAtgGTCGAGCAG | pWX995 |
| oWX2597 | CGCCAAGCTTCTCTGCAGGATATCccagggatggcattaaggtcc | sequencing |
| oWX2600 | AGCGAATTCGTGGATCCAGATATCcttcgccaacgtgctggatgcc | sequencing |
| oWX2649 | AAgctTacataaggaggaactactatgAGTAAAGGAGAAGAACTTTTCAC | BWX5309 |
| oWX2650 | cagctatgacaaacaaatgaaacagc | BWX5309, BWX5329 |
| oWX2651 | ggatgccgatacggctgaagcg | BWX5309, BWX2651 |
| oWX2655 | atagtagttcctccttatgtAAGCTTAATTGTTATCCGCTCAC | BWX5309 |
| oWX2668 | TGCCTCAAGCTAGAGAGTCgatgttcagacgctcagcttcag | BWX5309 |
| oWX2669 | gaagctgagcgtctgaacatcGACTCTCTAGCTTGAGGCATC | BWX5309 |
| oWX2674 | CCGAATTAGCTTGCATGCgatcactgcttggaccggtattcgg | BWX5329 |
| oWX2675 | aataccggtccaagcagtgatcGCATGCAAGCTAATTCGGTGG | BWX5329 |

**Table S1D. Next generation sequencing samples used in this study.**

| **Sample name** | **Figure** | **Reference** | **Identifier** |
| --- | --- | --- | --- |
| 401_Wang_HiC_AtWX063_ATGN | 3A, S4ACD | (2) | [GSM5542437](https://www.ncbi.nlm.nih.gov/geo/query/acc.cgi?acc=GSM5542437) |
| 408_Wang_HIC_AtWX089_ATGN | 3B, S4BCD | (2) | [GSM5542444](https://www.ncbi.nlm.nih.gov/geo/query/acc.cgi?acc=GSM5542444) |
| 443_Wang_HiC_AtWX283_ATGN | 3C, S4CD | This study | [GSM5870438](https://www.ncbi.nlm.nih.gov/geo/query/acc.cgi?acc=GSM5870438) |
| 444_Wang_HiC_AtWX110_ATGN | 3D, S4CD | This study | [GSM5870439](https://www.ncbi.nlm.nih.gov/geo/query/acc.cgi?acc=GSM5870439) |
| 445_Wang_HiC_AtWX121_ATGN | 3E, S4CD | This study | [GSM5870440](https://www.ncbi.nlm.nih.gov/geo/query/acc.cgi?acc=GSM5870440) |
| 446_Wang_HiC_AtWX286_ATGN | 3F, S4CD | This study | [GSM5870441](https://www.ncbi.nlm.nih.gov/geo/query/acc.cgi?acc=GSM5870441) |
| 447_Wang_ChIP_anti_mCherry_BWX5333_CH | 4CD | This study | [GSM5870442](https://www.ncbi.nlm.nih.gov/geo/query/acc.cgi?acc=GSM5870442) |
| 448_Wang_input_BWX5333_CH | 4CD | This study | [GSM5870443](https://www.ncbi.nlm.nih.gov/geo/query/acc.cgi?acc=GSM5870442) |
| 449_Wang_ChIP_anti_GFP_BWX5359_CH_20uMIPTG1h | 4CD | This study | [GSM5870444](https://www.ncbi.nlm.nih.gov/geo/query/acc.cgi?acc=GSM5870442) |
| 450_Wang_input_BWX5359_CH_20uMIPTG1h | 4CD | This study | [GSM5870445](https://www.ncbi.nlm.nih.gov/geo/query/acc.cgi?acc=GSM5870442) |
| 451_Wang_ChIP_anti_GFP_BWX5341_CH_20uMIPTG1h | 4C | This study | [GSM5870446](https://www.ncbi.nlm.nih.gov/geo/query/acc.cgi?acc=GSM5870442) |
| 452_Wang_input_BWX5341_CH_20uMIPTG1h | 4C | This study | [GSM5870447](https://www.ncbi.nlm.nih.gov/geo/query/acc.cgi?acc=GSM5870442) |
| 453_Wang_ChIP_anti_mCherry_BWX5353_CH_20uMIPTG1h | 4D | This study | [GSM5870448](https://www.ncbi.nlm.nih.gov/geo/query/acc.cgi?acc=GSM5870442) |
| 454_Wang_input_BWX5353_CH_20uMIPTG1h | 4D | This study | [GSM5870449](https://www.ncbi.nlm.nih.gov/geo/query/acc.cgi?acc=GSM5870442) |
| 458_Wang_ChIP_anti_GFP_AtWX234_ATGN_halfmMIPTG4h | S3A | This study | [GSM5870453](https://www.ncbi.nlm.nih.gov/geo/query/acc.cgi?acc=GSM5870442) |
| 459_Wang_input_AtWX234_ATGN_halfmMIPTG4h | S3A | This study | [GSM5870454](https://www.ncbi.nlm.nih.gov/geo/query/acc.cgi?acc=GSM5870442) |
| 460_Wang_ChIP_anti_GFP_AtWX236_ATGN_halfmMIPTG4h | S3C | This study | [GSM5870455](https://www.ncbi.nlm.nih.gov/geo/query/acc.cgi?acc=GSM5870442) |
| 461_Wang_input_AtWX236_ATGN_halfmMIPTG4h | S3C | This study | [GSM5870456](https://www.ncbi.nlm.nih.gov/geo/query/acc.cgi?acc=GSM5870442) |
| 462_Wang_ChIP_anti_GFP_AtWX265_ATGN_halfmMIPTG4h | S3B | This study | [GSM5870457](https://www.ncbi.nlm.nih.gov/geo/query/acc.cgi?acc=GSM5870442) |
| 463_Wang_input_AtWX265_ATGN_halfmMIPTG4h | S3B | This study | [GSM5870458](https://www.ncbi.nlm.nih.gov/geo/query/acc.cgi?acc=GSM5870442) |
| 464_Wang_ChIP_anti_GFP_AtWX289_LB_2mMTheo_1uMAHL_halfmMIPTG4h | S3D | This study | [GSM5870459](https://www.ncbi.nlm.nih.gov/geo/query/acc.cgi?acc=GSM5870442) |
| 465_Wang_input_AtWX289_LB_2mMTheo_1uMAHL_halfmMIPTG4h | S3D | This study | [GSM5870460](https://www.ncbi.nlm.nih.gov/geo/query/acc.cgi?acc=GSM5870442) |
| 466_Wang_ChIP_anti_GFP_AtWX289_LB_halfmMIPTG4h | S3D | This study | [GSM5870461](https://www.ncbi.nlm.nih.gov/geo/query/acc.cgi?acc=GSM5870442) |
| 467_Wang_input_AtWX289_LB_halfmMIPTG4h_4h | S3D | This study | [GSM5870462](https://www.ncbi.nlm.nih.gov/geo/query/acc.cgi?acc=GSM5870442) |
| 468_Wang_ChIP_anti_GFP_AtWX291_ATGN_halfmMIPTG4h | S3D | This study | [GSM5870463](https://www.ncbi.nlm.nih.gov/geo/query/acc.cgi?acc=GSM5870442) |
| 469_Wang_input_AtWX291_ATGN_halfmMIPTG4h | S3D | This study | [GSM5870464](https://www.ncbi.nlm.nih.gov/geo/query/acc.cgi?acc=GSM5870442) |
| 415_Wang_HiC_AtWX050_LB | S4E | (2) | [GSM5542451](https://www.ncbi.nlm.nih.gov/geo/query/acc.cgi?acc=GSM5542451) |
| 470_Wang_HiC_IB173_ATGN | S4F | This study | [GSM5870465](https://www.ncbi.nlm.nih.gov/geo/query/acc.cgi?acc=GSM5870465) |
| 471_Wang_HiC_IB172_ATGN | S4G | This study | [GSM5870466](https://www.ncbi.nlm.nih.gov/geo/query/acc.cgi?acc=GSM5870466) |
| 403_Wang_input_AtWX063_ATGN_rep2 | S5A-D | (2) | [GSM5542439](https://www.ncbi.nlm.nih.gov/geo/query/acc.cgi?acc=GSM5542439) |
| 404_Wang_ChIP_anti_AtParB_AtWX063_ATGN | S5AC | (2) | [GSM5542440](https://www.ncbi.nlm.nih.gov/geo/query/acc.cgi?acc=GSM5542440) |
| 405_Wang_ChIP_anti_AtRepBCh2_AtWX063_ATGN | S5BD | (2) | [GSM5542441](https://www.ncbi.nlm.nih.gov/geo/query/acc.cgi?acc=GSM5542441) |

**References**

1. Watson B, Currier TC, Gordon MP, Chilton MD, Nester EW. 1975. Plasmid required for virulence of Agrobacterium tumefaciens. J Bacteriol 123:255-64.

2. Ren Z, Liao Q, Karaboja X, Barton IS, Schantz EG, Mejia-Santana A, Fuqua C, Wang X. 2022. Conformation and dynamic interactions of the multipartite genome in Agrobacterium tumefaciens. Proc Natl Acad Sci U S A 119.

3. Dessaux Y, Tempe J, Farrand SK. 1987. Genetic analysis of mannityl opine catabolism in octopine-type Agrobacterium tumefaciens strain 15955. Mol Gen Genet 208:301-8.

4. Ireton K, Gunther NWt, Grossman AD. 1994. spo0J is required for normal chromosome segregation as well as the initiation of sporulation in Bacillus subtilis. J Bacteriol 176:5320-9.

5. Graham TG, Wang X, Song D, Etson CM, van Oijen AM, Rudner DZ, Loparo JJ. 2014. ParB spreading requires DNA bridging. Genes Dev 28:1228-38.

6. Wang X, Le TB, Lajoie BR, Dekker J, Laub MT, Rudner DZ. 2015. Condensin promotes the juxtaposition of DNA flanking its loading site in Bacillus subtilis. Genes Dev 29:1661-75.

7. Brandao HB, Ren Z, Karaboja X, Mirny LA, Wang X. 2021. DNA-loop-extruding SMC complexes can traverse one another in vivo. Nat Struct Mol Biol 28:642-651.

8. Nielsen HJ, Ottesen JR, Youngren B, Austin SJ, Hansen FG. 2006. The Escherichia coli chromosome is organized with the left and right chromosome arms in separate cell halves. Mol Microbiol 62:331-8.

9. Wagner JK, Marquis KA, Rudner DZ. 2009. SirA enforces diploidy by inhibiting the replication initiator DnaA during spore formation in Bacillus subtilis. Mol Microbiol 73:963-74.

10. Zupan JR, Grangeon R, Robalino-Espinosa JS, Garnica N, Zambryski P. 2019. GROWTH POLE RING protein forms a 200-nm-diameter ring structure essential for polar growth and rod shape in Agrobacterium tumefaciens. Proc Natl Acad Sci U S A 116:10962-10967.

11. Karimova G, Pidoux J, Ullmann A, Ladant D. 1998. A bacterial two-hybrid system based on a reconstituted signal transduction pathway. Proc Natl Acad Sci U S A 95:5752-6.

12. Hinz AJ, Larson DE, Smith CS, Brun YV. 2003. The Caulobacter crescentus polar organelle development protein PodJ is differentially localized and is required for polar targeting of the PleC development regulator. Mol Microbiol 47:929-41.

13. Khan SR, Gaines J, Roop RM, 2nd, Farrand SK. 2008. Broad-host-range expression vectors with tightly regulated promoters and their use to examine the influence of TraR and TraM expression on Ti plasmid quorum sensing. Appl Environ Microbiol 74:5053-62.

14. Figueroa-Cuilan W, Daniel JJ, Howell M, Sulaiman A, Brown PJ. 2016. Mini-Tn7 Insertion in an Artificial attTn7 Site Enables Depletion of the Essential Master Regulator CtrA in the Phytopathogen Agrobacterium tumefaciens. Appl Environ Microbiol 82:5015-25.

15. Wang X, Montero Llopis P, Rudner DZ. 2014. Bacillus subtilis chromosome organization oscillates between two distinct patterns. Proc Natl Acad Sci U S A 111:12877-82.
