# Supplementary material for: Centromere Interactions Promote the Maintenance of the Multipartite Genome in Agrobacterium tumefaciens
Source: mBio. 2022 May 10;13(3):e00508-22. doi: 10.1128/mbio.00508-22 (PMC9239152; doi:10.1128/mbio.00508-22)
Supplement: TEXT S1 [file mbio.00508-22-s0008.docx]

**SUPPLEMENTAL MATERIALS AND METHODS**

**Plasmid construction**

**pWX588** [*ycgO::Pspank* (optRBS) gfp-spo0J (parS*)* (*cat*)] was constructed by a ligation reaction containing two DNA fragments: 1) pAM12 (1) was digested by XmaI and NheI to give *ycgO::Pspank* cat*; 2) *(optRBS) gfp-spo0J (parS*)* was amplified using oWX998 and oWX999 from pKM256 (2).

**pWX822** [pSRKKm *msfgfp-popZ* (Atu1720/ATU_RS08420) (kan)] was constructed by an isothermal assembly reaction containing three DNA fragments: 1) pSRKKm digested by NdeI and HindIII; 2) *msfgfp* amplified using oWX2044 and oWX2046 from pSRKKm *msfgfp*; 3) *At popZ* amplified using oWX2051 and oWX2052 from C58 genomic DNA. The construct was sequenced using oWX1835, oWX2060 and oWX2061.

**pWX839** [pNPTS138 ∆*popZ* (Atu1720/ATU_RS08420) (*kan*)] was constructed by an isothermal assembly reaction containing three gel-purified fragments: 1) pNPTS138 digested by EcoRV; 2) *At popZ* upstream region amplified using oWX2160 and oWX2161 from C58 genomic DNA; 3) *At popZ* downstream region amplified using oWX2162 and oWX2163 from C58 genomic DNA. The construct was sequenced using oWX1854 and oWX1855.

**pWX845** [pKT25 *t25-parB1* (Atu2828/ATU_RS13770) (*kan*)] was constructed by ligating two DNA fragments: 1) pKT25 digested by XbaI and XmaI; 2) *At parB1* amplified using oWX2202 and oWX2193 from C58 gDNA and then digested by XbaI and XmaI. The construct was sequenced using oWX1789 and oWX1790.

**pWX846** [pKT25 *t25-repB^Ch2^* (Atu3923/ATU_RS18280) (*kan*)] was constructed by ligating two DNA fragments: 1) pKT25 digested by XbaI and XmaI; 2) *At repB^Ch2^* amplified using oWX2203 and oWX2197 from C58 gDNA and then digested by XbaI and XmaI. The construct was sequenced using oWX1789 and oWX1790.

**pWX847** [pKNT25 *parB1-t25* (Atu2828/ATU_RS13770) (*kan*)] was constructed by ligating two DNA fragments: 1) pKNT25 digested by XbaI and XmaI; 2) *At parB1* amplified using oWX2190 and oWX2191 from C58 gDNA and then digested by XbaI and XmaI. The construct was sequenced using oWX1782 and oWX1783.

**pWX848** [pKNT25 *repB^Ch2^-t25* (Atu3923/ATU_RS18280) (*kan*)] was constructed by ligating two DNA fragments: 1) pKT25 digested by XbaI and XmaI; 2) *At repB^Ch2^* amplified using oWX2194 and oWX2195 from C58 gDNA and then digested by XbaI and XmaI. The construct was sequenced using oWX1782 and oWX1783.

**pWX849** [pKT18 *t18-parB1* (Atu2828/ATU_RS13770) (*amp*)] was constructed by ligating two DNA fragments: 1) pKT18 digested by XbaI and XmaI; 2) *At parB1* amplified using oWX2202 and oWX2193 from C58 gDNA and then digested by XbaI and XmaI. The construct was sequenced using oWX1789 and oWX1790.

**pWX850** [pKT18 *t18-repB^Ch2^* (Atu3923/ATU_RS18280) (*amp*)] was constructed by ligating two DNA fragments: 1) pKT18 digested by XbaI and XmaI; 2) *At repB^Ch2^* amplified using oWX2203 and oWX2197 from C58 gDNA and then digested by XbaI and XmaI. The construct was sequenced using oWX1789 and oWX1790.

**pWX851** [pKNT18 *parB1-t18* (Atu2828/ATU_RS13770) (*amp*)] was constructed by ligating two DNA fragments: 1) pKNT18 digested by XbaI and XmaI; 2) *At parB1* amplified using oWX2190 and oWX2191 from C58 gDNA and then digested by XbaI and XmaI. The construct was sequenced using oWX1782 and oWX1783.

**pWX852** [pKNT18 *repB^Ch2^-t18* (Atu3923/ATU_RS18280) (*amp*)] was constructed by ligating two DNA fragments: 1) pKNT18 digested by XbaI and XmaI; 2) *At repB^Ch2^* amplified using oWX2194 and oWX2195 from C58 gDNA and then digested by XbaI and XmaI. The construct was sequenced using oWX1782 and oWX1783.

**pWX915** [pACYC *terminator* *Ppen*] was constructed by ligating two DNA fragments: 1) pWX294 digested by EcoRI and HindIII; 2) *Ppen* amplified using oWX2385 and oWX2386 from gWX46. pWX294 is an empty cloning vector with pACYC origin. Ppen is a constitutive promoter the penicillinase gene from *B. lycheniformis*. The construct was sequenced using oWX2395.

**pWX916** [pACYC *terminator* *Ppen* *cfp-parB^P1^-parS^P1^*] was constructed by an isothermal assembly reaction containing three gel-purified fragments: 1) pWX915 digested by HindIII and BamHI; 2) *rbs*-*cfp-parB^P1^* amplified using oWX2387 and oWX2388 from pFHC2973 (3); 3) *parS^P1^* amplified using oWX2389 and oWX2390 from gDNA of TND1379 (4). The construct was sequenced using oWX2395, oWX2396, oWX2397 and 2377.

**pWX930** [pNPTS138 *Ppen* *cfp-parB^P1^-parS^P1^* *kan* at Atu3054/ATU_RS14060] was constructed by an isothermal assembly reaction containing four gel-purified fragments: 1) pNPTS138 digested by EcoRV; 2) a part of Atu3054/ATU_RS14060 amplified using oWX2420 and oWX2421 from C58 gDNA; 3) *cfp-parB^P1^-parS^P1^* amplified using oWX2407 and oWX2408 from pWX916 4) a part of Atu3055/ATU_RS14065 amplified using oWX2422 and oWX2423 from C58 gDNA. The construct was sequenced using oWX2424, oWX2426, oWX2377 and oWX2425.

**pWX936** [pNPTS138 *PT7strong* *cfp-parB^P1^-parS^P1^* at Atu3054/ATU_RS14060] was constructed by an isothermal assembly reaction containing one gel-purified fragments: pWX930 backbone amplified using oWX2431 and oWX2432. The construct was sequenced using oWX2424, oWX2426, oWX2377 and oWX2425.

**pWX962** [pNPTS138 *PT7strong cfp-parB^P1^-parS^P1^* at Atu0048/ATU_RS00235] was constructed by an isothermal assembly reaction containing four gel-purified fragments: 1) pNPTS138 digested by EcoRV; 2) *PT7strong* *cfp-parB^P1^-parS^P1^* amplified using oWX2407 and oWX2408 from pWX936; 3) a part of Atu0047/ATU_RS00230 amplified using oWX2502 and oWX2503 from C58 gDNA; 4) a part of Atu0048/ATU_RS00235 amplified using oWX2504 and oWX2505 from C58 gDNA. The construct was sequenced using oWX2506, oWX2377, oWX2426, oWX2507.

**pWX995** [pNPTS138 terminators *PT7strong mcherry-parB^P1^-parS^P1^* at Atu0048/ATU_RS00235] was constructed by an isothermal assembly reaction containing two gel-purified fragments: 1) pWX962 backbone amplified using oWX2589 and oWX2590 on pWX962; 2) *mcherry* amplified using oWX2584 and oWX2585 from gDNA of BWX2208 (5). The construct was sequenced using oWX2506, oWX2377, oWX2426 and oWX2507.

**pIB315** [pNPTS138 15955 ∆*popZ* (ISGA_1749) (*kan*)] was constructed in two steps. First, *At* 15955 *popZ* upstream amplified using IPB140 and IBP141 and *At* 15955 *popZ* downstream amplified using IPB142 and IBP143 from 15955 gDNA were stitched together by PCR and then ligated into pGEM T-easy (Promega), confirmed by sequencing (6). Next the stitched fragment digested using BamH1 and Nhe1 and pNPTS138 digested with the same enzymes were ligated together.

**pIB316** [pNPTS138 15955 ∆*podJ* (ISGA_411) (*kan*)] was constructed in two steps. First, *At* 15955 *podJ* upstream amplified using IPB146 and IBP147 and *At* 15955 *podJ* downstream amplified using IPB148 and IBP149 from 15955 gDNA were stitched together by PCR and then ligated into pGEM T-easy (Promega), confirmed by sequencing (6). Next the stitched fragment digested using BamH1 and Nhe1 and pNPTS138 digested with the same enzymes were ligated together.

***A. tumefaciens* Strain construction**

In general, in-frame deletions of C58 *A. tumefaciens* strains were constructed using a previously described allelic replacement method (6). Briefly, regions flanking the gene to be deleted were PCR amplified using Phusion (NEB M0530) or Q5 polymerase (NEB M0491) and cloned into pNPTS138 (7), a ColE1 suicide plasmid that confers kanamycin resistance and sucrose sensitivity, by isothermal assembly reactions. See Plasmid construction for details. pNPTS138 deletion constructs were then introduced into *A. tumefaciens* C58 via mating with *E. coli* S17-1/λpir (8) carrying the appropriate construct. Screening for plasmid integration and target gene deletion was performed as previously described (6, 9). Colony PCR was used to amplify the region to confirm the deletion mutants. Specifically,

**C58*, mcherry-parB^P1^-parS^P1^* inserted between Atu0047/ATU_RS00230 and Atu0048/ATU_RS00235, 50 kb from *ori1*, *ygfp-parB^pMT1^-parS^pMT1^* inserted between Atu3973/ATU_RS18530 and Atu3974/ATU_RS18535, 57 kb from *ori2* (AtWX356)** was generated in two steps. First, pWX967 was used to insert *ygfp-parB^MT1^-parS^MT1^* between Atu3973/ATU_RS18530 and Atu3974/ATU_RS18535, 57 kb from *ori2*, generating AtWX295. This strain was confirmed using oWX2508 and oWX2511. Next, pWX995 was used to insert *mcherry-parB^P1^-parS^P1^* between Atu0047/ATU_RS00230 and Atu0048/ATU_RS00235, 50 kb from *ori1*, generating AtWX356. This strain was confirmed using oWX2502 and oWX2505.

**C58*, ∆repB^Ch2^*, *mcherry-parB^P1^-parS^P1^* inserted between Atu0047/ATU_RS00230 and Atu0048/ATU_RS00235, 50 kb from *ori1*, *ygfp-parB^pMT1^-parS^pMT1^* inserted between Atu3973/ATU_RS18530 and Atu3974/ATU_RS18535, 57 kb from *ori2* (AtWX402)** was generating using pWX854 on AtWX356 (see above), and conformed using oWX2076 and oWX2077.

**C58,** ***∆traI*, *tetRA*::*gen* *PtraI-riboswitch-parB1*(Atu2828/ATU_RS13770) *traR*, *mcherry-parB^P1^-parS^P1^* inserted between Atu0047/ATU_RS00230 and Atu0048/ATU_RS00235, 50 kb from *ori1*, *ygfp-parB^pMT1^-parS^pMT1^* inserted between Atu3973/ATU_RS18530 and Atu3974/ATU_RS18535, 57 kb from *ori2* (AtWX496)** was generated in two steps. First, pWX967 was used to insert *ygfp-parB^MT1^-parS^MT1^* between Atu3973/ATU_RS18530 and Atu3974/ATU_RS18535, 57 kb from *ori2* on AtWX192 (10), generating AtWX486. This strain was confirmed using oWX2508 and oWX2511. Next, pWX995 was used to insert *mcherry-parB^P1^-parS^P1^* between Atu0047/ATU_RS00230 and Atu0048/ATU_RS00235, 50 kb from *ori1* on AtWX486, generating AtWX496. This strain was confirmed using oWX2502 and oWX2505. AtWX192 contains *∆traI*, *tetRA*::*gen* *PtraI-riboswitch-parB1*(Atu2828/ATU_RS13770) *traR* (10).

**C58, *∆traI*, *tetRA*::*gen* *PtraI-riboswitch-parB1*(Atu2828/ATU_RS13770) *traR*, *ygfp-parB^pMT1^-parS^pMT1^* inserted between Atu5336/ATU_RS25500 and Atu5337/ATU_RS25505, 11 kb from *oAt* (AtWX498)** was generated using pWX1005 on AtWX192 (10), and confirmed using oWX2597 and oWX2600.

**C58, *∆repB^Ch2^*, *ygfp-parB^pMT1^-parS^pMT1^* inserted between Atu5336/ATU_RS25500 and Atu5337/ATU_RS25505, 11 kb from *oAt* (AtWX500)** was generated using pWX1005 on AtWX089 (10), and confirmed using oWX2597 and oWX2600. AtWX089 contains *∆repB^Ch2^* (10).

**C58, *∆podJ* (Atu0499/ATU_RS02460) (AtWX283)** was generated using *pGM9*, and confirmed using oWX2291 and oWX2292.

**C58, *∆popZ* (Atu1720/ATU_RS08420) (AtWX110)** was generated using pWX839, and confirmed using oWX2160 and oWX2163.

**C58, *∆popZ* (Atu1720/ATU_RS08420) *∆podJ* (Atu0499/ATU_RS02460) (AtWX121)** was generated using pGM9 on AtWX110, and confirmed using oWX2291 and oWX2292.

**C58, *∆gpr* (Atu1348/ATU_RS06650) (AtWX286)** was generated using pJZ298 (11), and confirmed using oWX2530 and oWX2531.

**15955,** ***∆podJ* (ISGA_411) (IB172)** was generated using pIB316, and confirmed using IBP144 and IBP145.

**15955,** ***∆popZ* (ISGA_1749) (IB173)** was generated using pIB315, and confirmed using IBP150 and IBP151.

Replicative plasmids were introduced to *A. tumefaciens* by electroporation as previously described (6). pWX822, pJZ253, pMAT3 were electroporated into C58 WT, generating AtWX234, AtWX236, AtWX265. pWX970 was electroporated into AtWX110, AtWX121, AtWX283 and AtWX286 to generate AtWX303, AtWX305, AtWX307 and AtWX309. pWX822 was electroporated into AtWX089 (10) and AtWX192 (10), generating AtWX291 and AtWX289, respectively.

***B. subtilis* Strain construction**

***pelB::Psoj* *mCherry-parB1_At_* *tet* (BWX5258)** A ligation reaction containing the following two DNA fragments was directly transformed to PY79: 1) pWX564 [*pelB::Psoj-mcherry-spo0J (parS*) (tet)*] (12) cut with BamHI and XhoI to remove *spo0J (parS*)*; 2) *parB1_At_* (amplified from C58 genomic DNA using oWX2563 and oWX2564, and then cut with BamHI and XhoI). The transformants were amplified using oWX776 and oML85 and sequenced using oWX776 and oML85.

***ycgO::Psoj mgfpmut3-RepB^Ch2^_At_ cat* (BWX5260)** A ligation reaction containing the following three DNA fragments was directly transformed to PY79: 1) an empty cloning vector pKM077 [*ycgO::cat*] cut with EcoRI and BamHI; 2) *repB^Ch2^_At_* (amplified from C58 genomic DNA using oWX2566 and oWX2567, and then cut with BamHI and XhoI); 3) *Psoj mgfpmut3* liborated from pWX563 using EcoRI and XhoI. The transformants were amplified and sequenced using oWX2497 and oWX2568. pWX563 (13) contains *pelB::Psoj-mgfpmut3-spo0J (parS*) tet*.

***parS2_At_* cluster at -91° *kan* (BWX5265)** An isothermal assembly reaction containing the following three PCR products was directly transformed to PY79: 1) the region containing *ytuF* upstream region (amplified from PY79 genomic DNA using oWX1279 and oWX2569); 2) the *parS2_At_* region (amplified from C58 genomic DNA using oWX2570 and oWX2571); 3) the region containing *kan*, *ytuF* and *ytuF* downstream (amplified from BWX3379 genomic DNA (14) using primers oWX439 and oWX1282). The transformants were amplified using oWX1283 and oML83 and sequenced using oWX1283 and oML83.

***parS∆9 no a.b., ycgO::Phyperspank-optRBS-mgfpmut3- repB^Ch2^_At_ cat* (BWX5309)** An isothermal assembly reaction containing the following three PCR products was directly transformed to BWX3212 (15): 1) the region containing *ycgO* downstream (amplified from PY79 genomic DNA using oWX2668 and oWX2650); 2) the *Phyperspank* promotor amplified from pJW005 (16) using oWX2655 and oWX2669); 3) the region containing *mgfpmut3-repB^Ch2^_At_*, *cat*, *ycgO* downstream (amplified from genomic DNA of BWX5260 using primers oWX2649 and oWX2651). The transformants were amplified using oWX2568 and oWX2560 and sequenced using oWX2568 and oWX2497.

***ycgO::Phyperspank* *mgfpmut3-repB^Ch2^*_At_ *cat*, *parS∆9* (BWX5329)** An isothermal assembly reaction containing the following three PCR products was directly transformed to BWX3212 (15): 1) the region containing *ycgO* downstream and *Phyperspank* *mgfpmut3-repB^Ch2^*_At_ (amplified from genomic DNA of BWX5309 (see above) using oWX2674 and oWX2650); 2) the *lacI-cat* and *ycgO* upstream (amplified from pWX588 using oWX2675 and oWX2651). pWX588 contains *ycgO::Pspank* (optRBS) gfp-spo0J (parS*) cat*. The transformants were amplified using oWX2568 and oWX2560 and sequenced using oWX2568 and oWX2497.

After individual *B. subtlis* constructs were built as above, their genomic DNA was extracted and used in successive transformations to build BWX5333, BWX5341, BWX5349, BWX5353, BWX5359.

**References**

1. Meeske AJ, Riley EP, Robins WP, Uehara T, Mekalanos JJ, Kahne D, Walker S, Kruse AC, Bernhardt TG, Rudner DZ. 2016. SEDS proteins are a widespread family of bacterial cell wall polymerases. Nature 537:634-638.

2. Sullivan NL, Marquis KA, Rudner DZ. 2009. Recruitment of SMC by ParB-parS organizes the origin region and promotes efficient chromosome segregation. Cell 137:697-707.

3. Nielsen HJ, Ottesen JR, Youngren B, Austin SJ, Hansen FG. 2006. The Escherichia coli chromosome is organized with the left and right chromosome arms in separate cell halves. Mol Microbiol 62:331-8.

4. Dalia AB, Dalia TN. 2019. Spatiotemporal Analysis of DNA Integration during Natural Transformation Reveals a Mode of Nongenetic Inheritance in Bacteria. Cell 179:1499-1511 e10.

5. Wang X, Tang OW, Riley EP, Rudner DZ. 2014. The SMC condensin complex is required for origin segregation in Bacillus subtilis. Curr Biol 24:287-92.

6. Morton ER, Fuqua C. 2012. Laboratory maintenance of Agrobacterium. Curr Protoc Microbiol Chapter 1:Unit3D 1.

7. Hinz AJ, Larson DE, Smith CS, Brun YV. 2003. The Caulobacter crescentus polar organelle development protein PodJ is differentially localized and is required for polar targeting of the PleC development regulator. Mol Microbiol 47:929-41.

8. Simon RP, U; Pühler, Alfred. 1983. A Broad Host Range Mobilization System for In Vivo Genetic Engineering: Transposon Mutagenesis in Gram Negative Bacteria. Nature Biotechnology 1:pages 784–791.

9. Barton IS, Platt TG, Rusch DB, Fuqua C. 2019. Destabilization of the Tumor-Inducing Plasmid from an Octopine-Type Agrobacterium tumefaciens Lineage Drives a Large Deletion in the Co-resident At Megaplasmid. G3 (Bethesda) 9:3489-3500.

10. Ren Z, Liao Q, Karaboja X, Barton IS, Schantz EG, Mejia-Santana A, Fuqua C, Wang X. 2022. Conformation and dynamic interactions of the multipartite genome in Agrobacterium tumefaciens. Proc Natl Acad Sci U S A 119.

11. Zupan JR, Grangeon R, Robalino-Espinosa JS, Garnica N, Zambryski P. 2019. GROWTH POLE RING protein forms a 200-nm-diameter ring structure essential for polar growth and rod shape in Agrobacterium tumefaciens. Proc Natl Acad Sci U S A 116:10962-10967.

12. Wang X, Montero Llopis P, Rudner DZ. 2014. Bacillus subtilis chromosome organization oscillates between two distinct patterns. Proc Natl Acad Sci U S A 111:12877-82.

13. Graham TG, Wang X, Song D, Etson CM, van Oijen AM, Rudner DZ, Loparo JJ. 2014. ParB spreading requires DNA bridging. Genes Dev 28:1228-38.

14. Brandao HB, Ren Z, Karaboja X, Mirny LA, Wang X. 2021. DNA-loop-extruding SMC complexes can traverse one another in vivo. Nat Struct Mol Biol 28:642-651.

15. Wang X, Le TB, Lajoie BR, Dekker J, Laub MT, Rudner DZ. 2015. Condensin promotes the juxtaposition of DNA flanking its loading site in Bacillus subtilis. Genes Dev 29:1661-75.

16. Wagner JK, Marquis KA, Rudner DZ. 2009. SirA enforces diploidy by inhibiting the replication initiator DnaA during spore formation in Bacillus subtilis. Mol Microbiol 73:963-74.
